# Hs27 fibroblast response to contact guidance cues

**DOI:** 10.1038/s41598-023-48913-9

**Published:** 2023-12-07

**Authors:** C. Kim, M. Robitaille, J. Christodoulides, Y. Ng, M. Raphael, W. Kang

**Affiliations:** 1 Mechanical Engineering, School for Engineering of Matter, Transport and Energy, Arizona State University, Tempe, AZ 85281 USA; 2 US Naval Research Laboratory, Washington, DC 20375 USA

**Keywords:** Engineering, Biomedical engineering, Mechanical engineering, Cell biology, Cell migration, Cell polarity, Cellular imaging

## Abstract

Contact guidance is the phenomena of how cells respond to the topography of their external environment. The morphological and dynamic cell responses are strongly influenced by topographic features such as lateral and vertical dimensions, namely, ridge and groove widths and groove depth (${{\text{R}}}_{{\text{w}}}, {{\text{G}}}_{{\text{w}}},\,\mathrm{and}\,\,{{\text{G}}}_{{\text{D}}}$, respectively). However, experimental studies that independently quantify the effect of the individual dimensions as well as their coupling on cellular function are still limited. In this work, we perform extensive parametric studies in the dimensional space—well beyond the previously studied range in the literature—to explore topographical effects on morphology and migration of Hs27 fibroblasts via static and dynamic analyses of live cell images. Our static analysis reveals that the ${{\text{G}}}_{{\text{D}}}$ is most significant, followed by the ${{\text{R}}}_{{\text{w}}}$. The fibroblasts appear to be more elongated and aligned in the groove direction as the ${{\text{G}}}_{{\text{D}}}$ increases, but their trend changes after 725 nm. Interestingly, the cell shape and alignment show a very strong correlation regardless of ${{\text{G}}}_{{\text{D}}}$. Our dynamic analysis confirms that directional cell migration is also strongly influenced by the ${{\text{G}}}_{{\text{D}}}$, while the effect of the ${{\text{R}}}_{{\text{w}}}$ and ${{\text{G}}}_{{\text{w}}}$ is statistically insignificant. Directional cell migration, as observed in the static cell behavior, shows the statistically significant transition when the ${{\text{G}}}_{{\text{D}}}$ is 725 nm, showing the intimate links between cell morphology and migration. We propose possible scenarios to offer mechanistic explanations of the observed cell behavior.

## Introduction

Cellular responses to topographical cues, known as contact guidance, has been studied for several decades^1–3^ and the influence of topography on cell behaviors has been also investigated mainly from bio-chemical and -physical viewpoints. The fundamental understanding of the cell-substrate interactions can provide better insight into the complicated relations between cells and surrounding natural extracellular matrix (ECM)^4^. In addition, the underlying mechanisms in the cell-ECM relations become relevant for revealing key biomedical processes such as wound healing, cancer dissemination, tissue and organ formation and regeneration as well as development of next generation biomaterials^5–9^. Various studies performed using different types of cells show considerable changes in cell morphology and mobility in response to topographic cues^10–13^. Several techniques, such as nano/microfabricated grooves, functionalized surface patterns, and fibers in hydrogels^12,14–16^, have been developed and utilized to achieve well-controlled anisotropic topographical cues and quantify topography-dependent cell responses including cell morphology, movement, viability, proliferation and differentiation^13,16,17^.

Previous studies in the contact guidance field have shown that cellular anisotropic behavior takes place on a micro- and nano grooved substrate of varying dimensions^10,18–20^. In general, these studies show that (1) cells tend to elongate along the groove direction (i.e., the longitudinal direction of ridge and groove patterns) and (2) a degree of elongation and alignment is strongly influenced by lateral and vertical dimensions of the patterns (grooves and/or ridges). However, the results are still inconsistent and specific contributions from each topographic feature, namely, groove depth (${{\text{G}}}_{{\text{D}}}$), ridge (${{\text{R}}}_{{\text{w}}}$) and groove (${{\text{G}}}_{{\text{w}}}$) widths, are not fully characterized. As a result, there are controversial conclusions on which topographic feature is the most dominant effect on cellular behavior. For example, ${{\text{G}}}_{{\text{D}}}$ was identified as the dominant factor for cell elongation and alignment of baby hamster kidney (BHK) cells^10^ and corneal epithelial cells^11^. However, another study using rat dermal fibroblasts^19^ reported a considerable increase in the cell elongation and alignment ratios with a smaller ${{\text{R}}}_{{\text{w}}}$ and concluded that that ${{\text{R}}}_{{\text{w}}}$ is the most dominant dimension compared to ${{\text{G}}}_{{\text{w}}}$ and ${{\text{G}}}_{{\text{D}}}$.

Similar to the static cell behavior, dynamic cellular responses to contact guidance are cell specific. Neutrophils^21^, endothelial^22^, osteoblasts^23^, human cervical and lung cancer cells^24^ on patterned topographies show strongly directional and fast cell migration along the longitudinal direction of contact guidance patterns compared to control experiments on flat surfaces. However, the cell mobility of human aortic endothelial cells^22^ and corneal keratocytes^25^ are not strongly influenced by surface patterns. Such inconsistency in cell responses can be partially due to their cell type dependency as well as a lack of parametric studies for individual cues since the intrinsic morphology and/or variation of respective cell type in the intracellular population are closely related with their physicochemical functions^26^. Many studies have simultaneously varied two dimensions, ${{\text{R}}}_{{\text{w}}}\,\mathrm{and}\,{{\text{G}}}_{{\text{w}}}$, while poorly controlling over the other (${{\text{G}}}_{{\text{D}}}$), which can lead to inaccuracy in predicting cell behaviors because of inconclusive data generated by intertwined topographic effects.

Despite the inconsistency, it has been well accepted that contact guidance technologies considerably alter cell morphology^11,27,28^ and migration^25,28^ and, as a result, have been considered as a possible mechanism to enhance wound healing processes, e.g., by guiding cells to wound sites and controlling their elongation, alignment, and directionality. In this regard, fibroblasts become relevant due to their important roles in wound healing process including breaking down fibrin clots and repairing ECM and collagen structures^29^. However, experimental characterizations of anisotropic morphological behaviors of fibroblasts on contact guidance surfaces are still limited to a relatively small range of topographical dimensions, i.e., G_w_ and R_w_ $\le 2\,\mathrm{ \mu m}$ or G_D_ $\le 300\,\mathrm{ nm}$^11,25,28^. Furthermore, the effect of contact guidance on dynamic cell behavior has been rarely performed and, to our best knowledge, the available results are limited to G_w_ and R_w_ $\le 2\,\mathrm{ \mu m}$ with G_D_ $\le 300\,\mathrm{ nm}$ and G_w_ and R_w_ $=3, 5,\,\mathrm{and}\,10\,\mathrm{ \mu m}$ with G_D_ $\le 300\,\mathrm{ nm}$^25,28^. However, considering typical dimensions (diameter > $10 \,\mathrm{\mu m}$ and thickness $\sim 550 {\text{ nm}}$) of fibroblasts^30^, it is worth investigating cell anisotropic behaviors on the substrate having intermediate feature sizes of widths and depths ($2\,\mathrm{ \mu m}\le$ G_w_ and R_w_, and $330\,\mathrm{ nm}\le$ G_D_ $\le 1000\,\mathrm{ nm}$) since each fibroblast can spread on enough number of topographical cues to impose constraints along the groove direction, which is sufficient to affect their morphology and migration.

Beyond the necessity of a contact guidance study for fibroblasts with a broad range of topographical dimensions, it would be highly desirable to independently evaluate a contribution for individual topographic features. To our best knowledge, no effort has yet been made to perform a parametric study for fibroblasts in order to reveal each topographical effect, while holding one-dimension constant with varying the others. The quantitative characterization of cellular behaviors under influence of well controlled yet diverse combinations of geometrical cues is still challenging due to lack of a fabrication technique that allows precise control of small-scale topographic features and multiplexed cell studies under identical extracellular environments. Without such unique capabilities, characterizing the correlation between individual effects of topographical cues and the corresponding cellular behavior with statistical significance becomes non-trivial. On top of that, several hypotheses have been proposed to explain their underling mechanisms; some studies suggest that mechanical interlocking or capillary force results from the groove of the substrates^31,32^ and others present that cells lying on the micro- or nanotextured surfaces are able to reorganize their fibers network for balancing internal and external forces^33,34^. Characterizing the specific effects of individual topographical features, using a time-effective high throughput method, is crucial to explore possible mechanisms for cell-type dependent anisotropic cell behaviors and to provide effective design space for biomedical applications of contact guidance.

In here, we characterize the morphological and dynamic responses of Hs27 fibroblasts on multiplexed monolithic quartz chips^35^ with a wide range of topographical dimensions well beyond what has been previously reported. Our multiplexed approach allows quantitative characterizations of ${{\text{R}}}_{{\text{w}}}, {{\text{G}}}_{{\text{w}}},\,\mathrm{and}\,{{\text{G}}}_{{\text{D}}}$ dependent cell responses while minimizing unwanted biological variations during cell culture. First, we perform static analyses on three different morphological measurements, such as cell spread area, aspect ratio and cell alignment degree, due to their biological and biomedical relevance. Then, we quantify directional cell migration in the scope of effectively guided cell motion. Finally, we utilize both the cell membrane deformation and focal adhesion models to offer mechanistic explanations for the observed cell behavior of HS27 fibroblasts on the multiplexed contact guidance chips.

## Materials and methods

### Fabrication of platforms

**Figure 1:**
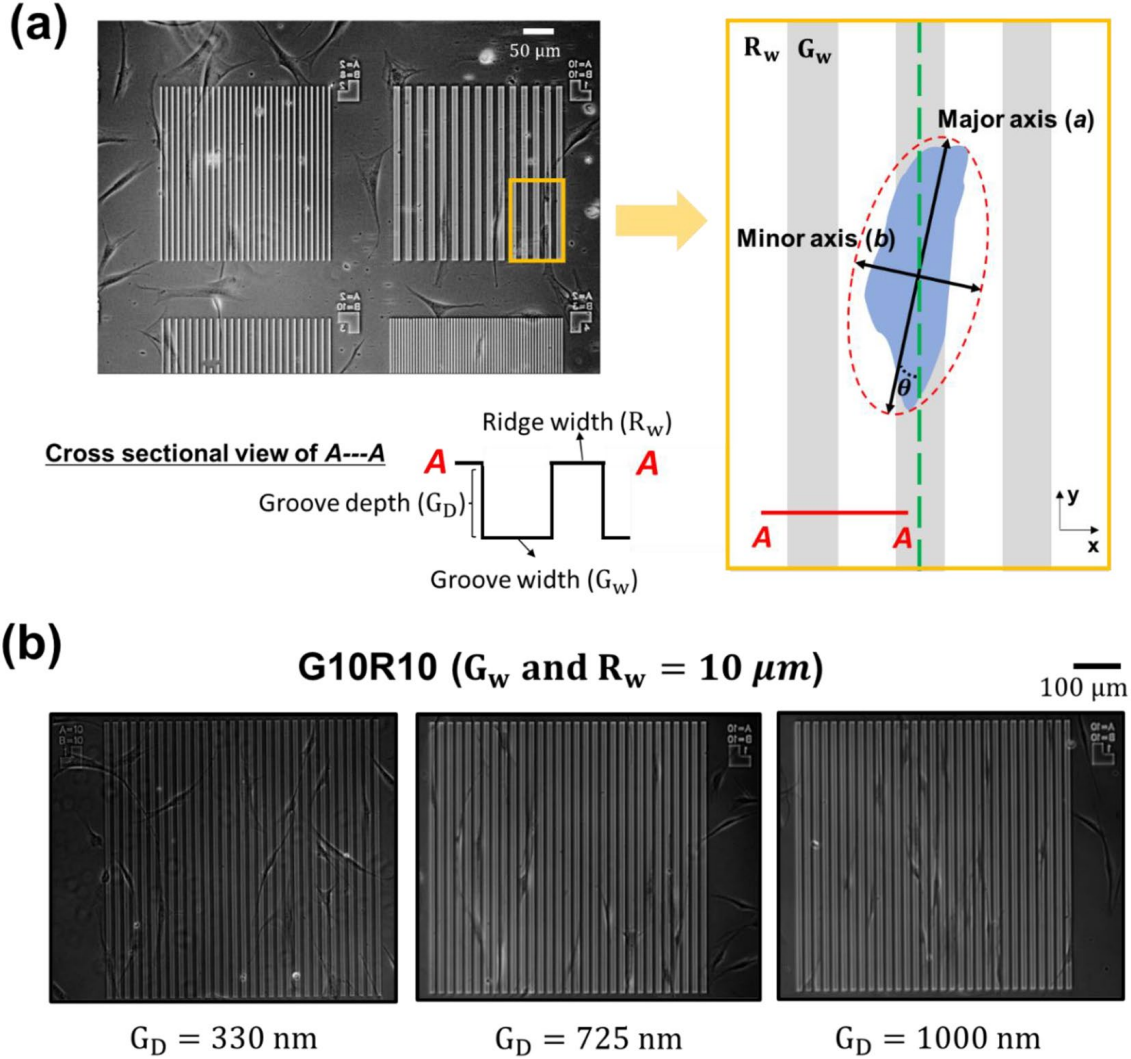
(**a**) A monolithic quartz contact guidance platform (left) and a schematic diagram of how cell topography is characterized on the micropatterned substrate (right). A–A is a cross sectional view of the platform showing groove (G_w_) and ridge (R_w_) widths, and groove depth (G_D_). The outline of a cell is fitted by an ellipse to estimate a length ratio of the major to minor axes and a cell alignment angle ($\uptheta$) between the major axis and the direction of the groove patterns. (**b**) Optical images of live Hs27 cells on ${{\text{G}}}_{{\text{w}}}={{\text{R}}}_{{\text{w}}}=10\,\upmu$m (i.e., G10R10) with (left) ${{\text{G}}}_{{\text{D}}}=$ 330 nm, (middle) = 725 nm, and (right) = 1000 nm, respectively.

To examine the effects of substrate anisotropy on cellular alignment, we created multiplexed patterns with an emphasis on achieving uniform smoothness on the entire substrate including the patterns. The surface topographical cues consist of ridge and groove widths, and groove depths using deep etching process and seeded Hs27 fibroblasts on the etched substrates as shown in Fig. 1a. To be specific, a 20 nm Cr thin film was e-beam evaporated onto 25 mm diameter fused silica coverslips and subsequently coated with AZ1518 photoresist (Microchemicals GmbH) and baked on a vacuum hot plate at 100 °C for 90 s. The photoresist was patterned in a Heidelberg VPG200++ laser pattern generator at a dose of 60 µC/cm^2^ and developed with the use of using AZ developer (Microchemicals GmbH) and rinsed in DI water. The patterned Cr film acted as hard mask, allowing only the exposed fused silica area to be etched (Oxford PlasmaLab 100 ICP380 system) by CHF_3_ and Ar, with flow rates of 10 and 15 sccm, respectively, at 3 mTorr and 20 °C to achieve various topographical conditions (more details are given in Ref.^35^). To systematically control topographical cues, ridge and groove widths (${{\text{R}}}_{{\text{w}}}$ and ${{\text{G}}}_{{\text{w}}}$, respectively) ranging from 2 to 10 μm with different ${{\text{R}}}_{{\text{w}}}$-to-${{\text{G}}}_{{\text{w}}}$ ratios were cofabricated on each multiplexed contact guidance chip. Three different groove depths (i.e., ${{\text{G}}}_{{\text{D}}}$ = 330, 725, and 1000 nm) were used to quantify the effect of groove depth on cell response. The detailed dimensions of the contact guidance chips are presented in Table S1. The lower limit of ${{\text{R}}}_{{\text{w}}}=2 \,\mathrm{\mu m}$ was motivated by the size of typical mature FAs^36,37^, whereas the upper limit of ridge or groove width is 10 μm, which is large enough not to confine focal adhesions and is also about the diameter of the nucleus^38^.

### Cell culture

For cell culture, Hs27 cells (ATCC, CRL-1634, Lot #7006836) were cultured in DMEM (ATCC, 30–2002) with 10% FBS (ATCC, 30-2020, Lot #802500) at 37 °C and 5% CO_2_ as per manufacturer instructions, without antibiotics. The contact guidance chips and surrounding chambers were assembled under aseptic conditions. Ethanol was introduced to the chambers for 5 min and then N_2_ dried for additional sterilization of the contact guidance chips. FN at 25 µg/mL in 10 mM PBS was drop coated directly onto the chips and allowed to adsorb for 1 h at room temperature, and subsequently rinsed 5× with DMEM to remove any excess FN. The contact guidance chambers were then allowed to equilibrate Zeiss microscopes featuring environmental chambers at 37 °C, 5% CO_2_ and 95% humidity for at least 30 min before the introduction of cells. Hs27 cells were harvested for contact guidance experiments between 30 and 60% confluence in their logarithmic growth phase. Complete media was aspirated and replaced with 0.25% Trypsin–EDTA (Gibco, #25200-056) for 5 min at 37 °C, after which serum free media was introduced.

### Data analysis

The morphology of Hs27 fibroblasts cultured on the monolithic quartz platform was evaluated by analyzing the shapes of all cells adhering to patterned geographies. Image analysis was conducted under 10× phase microscopy and those images were acquired every 10 min and converted into TIFF files using Zen software. For example, the optical images, taken 24 h after initial cell seeding using a 10× phase objective, in Fig. 1b shows Hs27 cells on G10R10 (${{\text{G}}}_{{\text{w}}}={{\text{R}}}_{{\text{w}}}=10 \,\mathrm{\mu m}$) with ${{\text{G}}}_{{\text{D}}}=330, 725,\,\mathrm{and}\,1000\,\mathrm{ nm}$ from left to right, respectively. The images show that the cells exhibit clearly different cell shapes depending on ${{\text{G}}}_{{\text{D}}}.$ By using ImageJ (version 1.41, NIH), we manually draw the outline of each cell on the substrates) and then, quantitatively measured the area of individual cells (see the Supplementary Fig. S1). The cell outline was then fitted using an ellipse to estimate the cell aspect ratio ($\,\mathrm{\alpha }$) using a length ratio of the major to minor axes and then obtain the cell alignment angle ($\theta$) between the major axis and the direction of groove patterns (see Fig. 1a).

The cell’s time-dependent coordinates were measured by tracking its nucleus via ManualTracking plugin in the ImageJ between 10 min time frames. For dynamic analysis, we only consider the following case: cells migrate more than 6 h and their nucleus are on the topographical structures, followed by Ref.^35^. This data has been used to compute several dynamic measurements (directional orientation, angular displacement, speed, mean square of distance, directionality ratio, and velocity autocorrelation). For all image analyses, we characterized more than 20 cells for each of the topographical dimensions including control experiments on a flat substrate. For precise analysis of cellular static behaviors, we ruled out the following two cases: (1) cells underwent mitosis and (2) cells spread over both flat and patterned areas of a substrate. Statistical analysis was performed by Kruskal Wallis nonparametric test and Dunn–Sidak post hoc test using MATLAB for the estimation of significance level and statistical comparison of each pair of groups. All data figures were prepared by MATLAB where the central mark indicates the median, and the bottom and top edges of the box point to the 25% and 75%, respectively. The outliers are plotted individually if they are more than ${{\text{q}}}_{3}+1.5\cdot \left({{\text{q}}}_{3}-{{\text{q}}}_{1}\right)$ or less than ${{\text{q}}}_{1}-1.5\cdot \left({{\text{q}}}_{3}-{{\text{q}}}_{1}\right)$, indicated by the ‘+’ symbol in which ${{\text{q}}}_{1} {\text{ and }} {{\text{q}}}_{3}$ are the 25th and 75th percentiles of the data set, respectively.

## Results

We experimentally characterize cell morphology and migration of Hs27 fibroblast on the monolithic quartz contact guidance platform. Our goal is to systematically quantify the effect of lateral (ridge width (${{\text{R}}}_{{\text{w}}}$), groove width (${{\text{G}}}_{{\text{w}}}$)), and vertical (groove depth (${{\text{G}}}_{{\text{D}}}$)) dimensions on static and dynamic behavior of fibroblast cells. For the lateral dimensions, we consider three different cases, namely, G2NR, GNR2, and GNRN. First, G2RN indicates fixed groove width (i.e., ${{\text{G}}}_{{\text{w}}}=2 \,\mathrm{\mu m}$) with different combinations of ridge width (i.e., ${{\text{R}}}_{{\text{w}}}$= $2, 3, 4, 6, 8,\,\mathrm{and}\,10 \,\mathrm{\mu m}$). Similarly, GNR2 corresponds to ${{\text{R}}}_{{\text{w}}}=2 \,\mathrm{\mu m}$ with different ${{\text{G}}}_{{\text{w}}}$ values (i.e., ${{\text{G}}}_{{\text{w}}}=2, 3, 4, 6,\,\mathrm{and}\,8\,\mathrm{ \mu m}$). G2RN and GNR2 are designed for independent characterization of the ${{\text{R}}}_{{\text{w}}}$- and ${{\text{G}}}_{{\text{w}}}$-dependent cell responses, respectively. Finally, GNRN represents topographical cues with ${{\text{R}}}_{{\text{w}}}/{{\text{G}}}_{{\text{w}}}=1$ where ${{\text{R}}}_{{\text{w}}}=2, 3, 4, 6, 8,\,\mathrm{and}\,10 \,\mathrm{\mu m}$ for exploring the ${{\text{G}}}_{{\text{w}}}$–${{\text{R}}}_{{\text{w}}}$ coupling effect. To reveal the effect of groove depth (${{\text{G}}}_{{\text{D}}}$), live cell experiments on G2NR, GNR2, and GNRN are performed and analyzed with three different groove depths (i.e., ${{\text{G}}}_{{\text{D}}}=330, 725,\,\mathrm{and}\,1000\,\mathrm{ nm}$).

### Cell morphology

In this section, three key cell morphological parameters (spread area (${A}_{cell}$), aspect ratio ($\alpha$), and alignment angle ($\theta$)) of fibroblast cells are quantified by performing image analyses of live cells on a contact guidance (see Fig. 1a).

Our image analysis on the cell spread area indicates that cells on the patterned substrate tend to have smaller spread areas compared those on the flat control substrate (see Fig. S2 in the Supplementary Document). As a reminder, image analysis techniques are discussed in “Materials and methods” section. The spread area (${A}_{Cell}$) on the control substrate (= $2816.8\,\upmu {{\text{m}}}^{2}$) is significantly larger than that on the topographical cues, but the variation in ${A}_{Cell}$ among different topographical cues is statistically insignificant (${A}_{Cell}=2365.3, 2115.3$ and $2259.4\,\mathrm{ \mu} {{\text{m}}}^{2}$ on ${G}_{D}=$ 330, 725, and 1000 nm, respectively). Consistent with our results, a similar trend was observed for NH3T3 fibroblast cells cultured on micropatterned polydimethylsiloxane (PDMS) substrate^39^.

**Figure 2:**
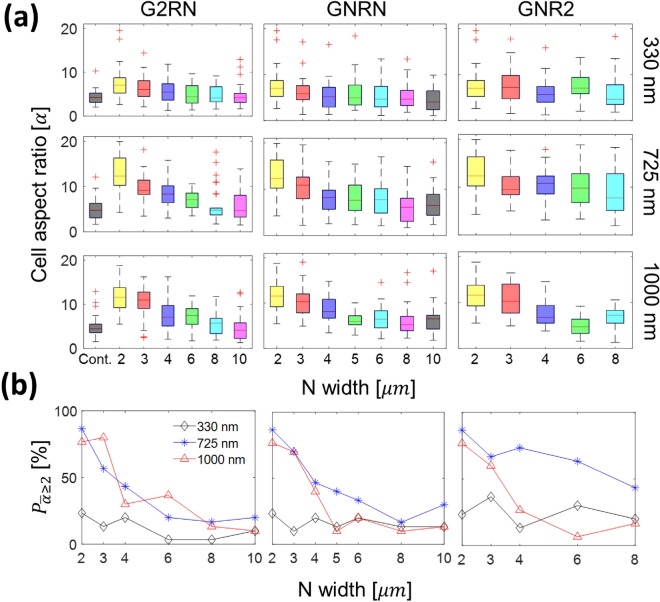
(**a**) A cell aspect ratio ($\mathrm{\alpha }$) for different topographic conditions. Each column represents three different cases: (from left to right) G2RN (${{\text{G}}}_{{\text{w}}}=2\,\mathrm{ \mu m}$ and ${{\text{R}}}_{{\text{w}}}=\,\mathrm{N \mu m}$), GNRN (${{\text{G}}}_{{\text{w}}}={{\text{R}}}_{{\text{w}}}=\,\mathrm{N \mu m}$), and GNR2 (${{\text{G}}}_{{\text{w}}}=\,\mathrm{N \mu m}$ and ${{\text{R}}}_{{\text{w}}}=2\,\mathrm{ \mu m}$). Each row indicates different groove depths: (from top to bottom) ${{\text{G}}}_{{\text{D}}}=330, 725,\,\mathrm{and}\,1000\,\mathrm{ nm}$ Cont. indicates a control experiment on a flat control substrate. (**b**) ${{{P}}}_{\overline{\mathrm{\alpha }}\ge 2 }$ is the percentage population of elongated cells ($\overline{\mathrm{\alpha } }\ge 2$) for (left to right) G2RN, GNRN, and GNR2, respectively.

Figure 2 shows the elongation of cells. More specifically, Fig. 2a summarizes the cellular aspect ratio ($\mathrm{\alpha }$) as a function of *N* (the size of lateral widths) for three different cases: G2RN (${{\text{G}}}_{{\text{w}}}=2\,\mathrm{ \mu m}$ and ${{\text{R}}}_{{\text{w}}}=\,\mathrm{N\ \mu m}$), GNRN (${{\text{G}}}_{{\text{w}}}={{\text{R}}}_{{\text{w}}}=\,\mathrm{N\ \mu m}$), and GNR2 (${{\text{G}}}_{{\text{w}}}=\,\mathrm{N\ \mu m}$ and ${{\text{R}}}_{{\text{w}}}=2\,\mathrm{ \mu m}$). In addition, different groove depths (${{\text{G}}}_{{\text{D}}}=330, 725,\,\mathrm{and}\,1000\,\mathrm{ nm}$) are considered. Note that *Cont.* indicates control experiments on flat control substrates. For clarification, the results of cell alignment and cell speed will be presented in the same way in the later sections to be consistent.

Consider the cellular aspect ratios from G2RN (see the first column in Fig. 2a). Two general trends are (1) $\mathrm{\alpha }$ decreases with increasing ${{\text{R}}}_{{\text{w}}}$ and (2) $\mathrm{\alpha }$ increases with increasing ${{\text{G}}}_{{\text{D}}}$. For instance, $\mathrm{\alpha }$ values of ${{\text{G}}}_{{\text{D}}}$ = 725 nm decrease from $\mathrm{\alpha }=$ 13.06 (G2R2) to $\mathrm{\alpha }=$ 5.81 (G2R10). Also, the distribution of the $\mathrm{\alpha }$ values is sensitive to ${{\text{G}}}_{{\text{D}}}$, e.g., a rate of decreasing $\mathrm{\alpha }$ with increasing ${{\text{R}}}_{{\text{w}}}$ is much faster for ${{\text{G}}}_{{\text{D}}}$ = 725 and 1000 nm compared to ${{\text{G}}}_{{\text{D}}}$ = 330 nm. The strong ${{\text{G}}}_{{\text{D}}}$ dependency in G2RN can be again validated by comparing the average aspect ratio (${\mathrm{\alpha }}_{{\text{ave}}}$) for each ${{\text{G}}}_{{\text{D}}}$. For example, ${\mathrm{\alpha }}_{{\text{ave}}}=8.66\,\mathrm{and}\,7.64$ for ${{\text{G}}}_{{\text{D}}}$ = 725 and 1000 nm, respectively, are significantly larger than ${\mathrm{\alpha }}_{{\text{ave}}}=5.92$ for ${{\text{G}}}_{{\text{D}}}$ = 330 nm and ${\mathrm{\alpha }}_{{\text{ave}}}=4.81$ for the flat substrate (control). Kruskal Wallis test (see Fig. S3 in the Supplementary) confirms that the effect of ${{\text{G}}}_{{\text{D}}}$ on $\mathrm{\alpha }$ is statistically significant (p << 0.001). The results of GNRN (second column) and GNR2 (third column) show somewhat similar qualitative trends of G2RN. However, our statistical test shown in Figs. S3 and S4 reveals that the p-values for varying ${{\text{R}}}_{{\text{w}}}$ are much smaller than those for ${{\text{G}}}_{{\text{w}}}$ regardless of ${{\text{G}}}_{{\text{D}}}$. The smaller p-values are mainly caused by more statistically significant pairs in each case (e.g., G2RN and GNR2) rather than small variance within each topography by comparing standard deviations of cell aspect ratio for varying ${{\text{R}}}_{{\text{w}}}$ and ${{\text{G}}}_{{\text{w}}}$. Therefore, we conclude that ${{\text{R}}}_{{\text{w}}}$ has stronger effect on the cell aspect ratio compared to ${{\text{G}}}_{{\text{w}}}$.

It is important to acknowledge that $\mathrm{\alpha }$ values depend on cell type as well as cell culture conditions. For example, different $\mathrm{\alpha }$ values have been reported for cells on flat control substrates, e.g., $\mathrm{\alpha }=$ 2–2.3 for NIH 3T3 fibroblasts and myofibroblasts^39,40^. To account for a natural tendency of such cell-type and cell-culture dependent behavior, here all the aspect ratios are normalized by the aspect ratio of cells on the control substrate (i.e., $\overline{\mathrm{\alpha } }=\,\mathrm{\alpha }/{\,\mathrm{\alpha }}_{{\text{Cont}}}$). Following the previous studies^39–41^, the percentage population (${P}_{\overline{\alpha }\ge 2}$) of elongated cells are quantified by using $\overline{\alpha }\ge 2$.

Figure 2b shows ${P}_{\overline{\alpha }\ge 2}$ values as a function of dimensional parameter *N* for cells on (left to right) G2RN, GNRN, and GNR2. In each plot, black diamonds, blue asterisks, and red triangles indicate ${{\text{G}}}_{{\text{D}}}$=330, 725, and 1000 nm, respectively. First, ${P}_{\overline{\alpha }\ge 2}$ for ${{\text{G}}}_{{\text{D}}}=330\,\mathrm{ nm}$ is much less sensitive to *N* compared to the deeper groove depths. As an example, due to the change in *N* from 2 to $8\,\mathrm{ \mu m}$, ${P}_{\overline{\alpha }\ge 2}$ of G2RN decreases from 86.7 to 16.7% on ${{\text{G}}}_{{\text{D}}}$ = 725 nm and from 76.7 to 13.3% on ${{\text{G}}}_{{\text{D}}}$ = 1000 nm, respectively. The similar trend can be seen in GNR2 as the corresponding ${P}_{\overline{\alpha }\ge 2}$ values are 86.7% (${{\text{G}}}_{{\text{D}}}$ = 725 nm) and 76.7% (${{\text{G}}}_{{\text{D}}}$ = 1000 nm) at ${\text{N}}=2\,\mathrm{ \mu m}$ and 43.3% and 16.7% at ${\text{N}}=8\,\mathrm{ \mu m}$, respectivtly. Clearly, ${P}_{\overline{\alpha }\ge 2}$ for ${{\text{G}}}_{{\text{D}}}=725\,\mathrm{and}\,1000\,\mathrm{ nm}$ decreases with an increase of *N* at a much faster rate compared to ${{\text{G}}}_{{\text{D}}}=330$ nm, which indicates a strong depth dependent cell behavior. To further consider the correlation between cell elongation and groove depth for all lateral dimensions (R_w_ and G_w_), the average ${P}_{\overline{\alpha }\ge 2}$ values for each depth, including all G2RN, GNRN, and GNR2 cases, are shown in see Fig. S5a. The average ${P}_{\overline{\alpha }\ge 2}$ values are 4.44%, 16.5%, 31.9%, and 25.0% on ${{\text{G}}}_{{\text{D}}}=0$ (control), 330, $725$, and 1000 nm, respectively.

We quantitatively characterize the effect of individual topographical features (R_w_, G_w_ and G_D_) and their products on ${P}_{\overline{\alpha }\ge 2}$ by conducting multiple linear regression analyses. Because ${P}_{\overline{\alpha }\ge 2}$ strongly depends on G_D_ and changes its trend between G_D_ = 725 nm and = 1000 nm, we performed the linear regression analyses twice using ${{\text{G}}}_{{\text{D}}}=0{-}725$ nm (Case 1) and ${{\text{G}}}_{{\text{D}}}=0{-}1000\, {\text{ nm}}$ (Case 2), respectively, as summarized in Table S2 (see the Supplementary Materials). For clarity, we use ${{\text{G}}}_{{\text{D}}}$, ${{\text{G}}}_{{\text{w}}}$, and ${{\text{R}}}_{{\text{w}}}=0$ for the flat substrates (control) as no topographical effect is expected on cell responses and, as a result, a constant term in the multiple linear regression model (see Eq. S1) becomes the ${P}_{\overline{\alpha }\ge 2}$ value for the control substrates. A few key conclusions are (1) ${R}^{2}$ values for Case 1 and 2 are significantly different (i.e., 0.835 for Case 1 and 0.612 for Case 2, respectively) and (2) despite the different ${R}^{2}$ values, the coefficient associated with ${{\text{G}}}_{{\text{D}}}$, for both cases, is 1–2 orders of magnitude larger than other coefficients with statistical significance (p < ${10}^{-8}$). The smaller ${R}^{2}$ value for Case 2 may confirm a change in the ${P}_{\overline{\alpha }\ge 2}$ trend for ${{\text{G}}}_{{\text{D}}}>725$ nm.

**Figure 3:**
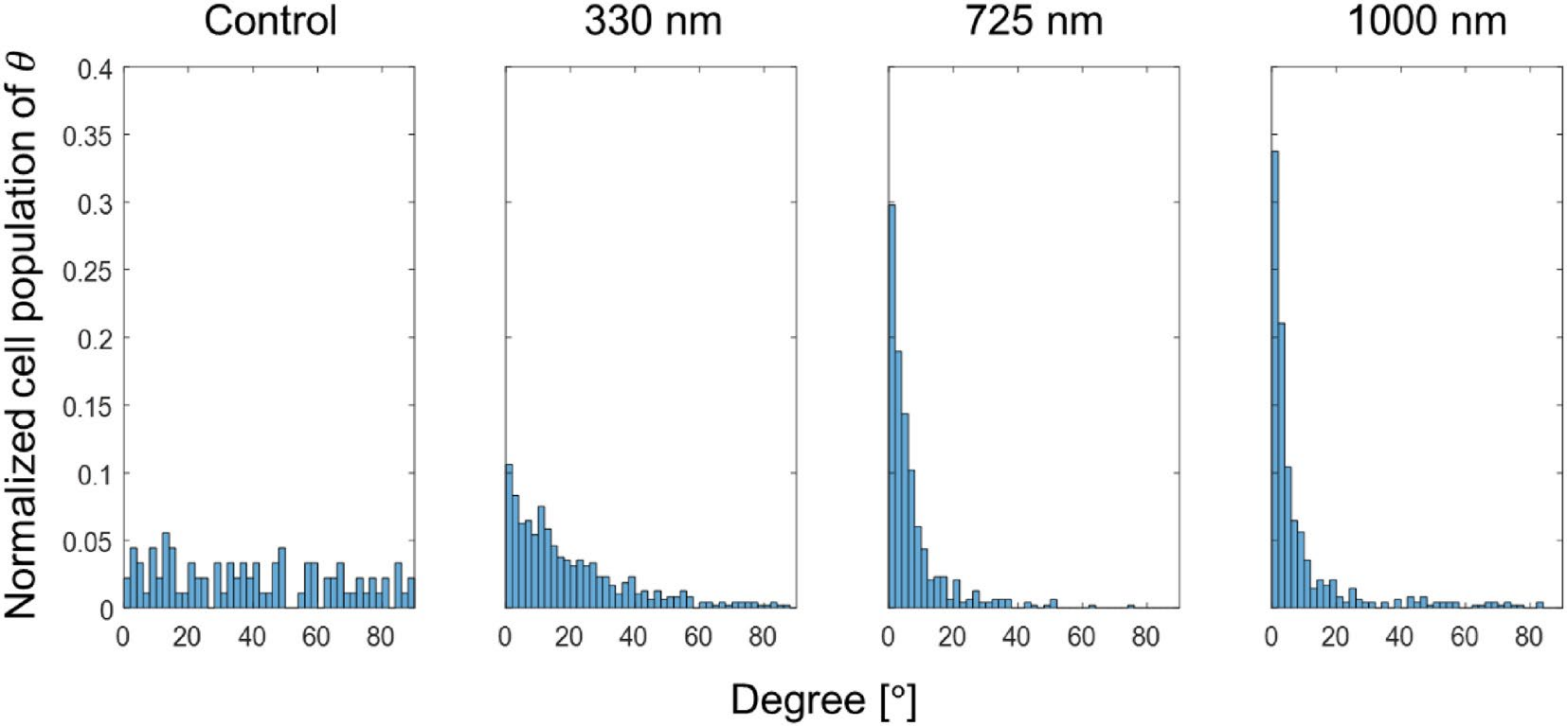
Normalized cell population as a function of cell alignment degree with respect to surface patterns: (from left to right) control, 330, 725, and 1000 nm. The bin size is $2^\circ$.

Finally, Fig. 3 shows the normalized cell population as a function of the cell alignment angle (*θ*) on the control and patterned substrates with three different groove depths (${{\text{G}}}_{{\text{D}}}$). Despite significantly different population distributions shown in the figure, the average values of the cell alignment angle (${\theta }_{ave}$) for ${{\text{G}}}_{{\text{D}}}=0 ({\text{control}}), 330, 725,$ and $1000\,\mathrm{ nm}$ are $39.68^\circ$, $19.70^\circ$, $7.23^\circ$, and $9.27^\circ$, respectively. For clarification, each average value for each ${{\text{G}}}_{{\text{D}}}$ includes G2RN, GNRN, and GNR2 and, therefore, the effect of ${{\text{G}}}_{{\text{D}}}$ is considered for all ${{\text{R}}}_{{\text{w}}}$ and ${{\text{G}}}_{{\text{w}}}$. Note $\theta$ for the patterned substrates are significantly smaller than that of the control substrate and their distributions are also statistically different with p-value << 0.001 as shown in see Fig. S6. The cells on the control substrates exhibit a uniform distribution of alignment angle distribution unlike the patterned substrates. This expected trend can be also seen in the linear increasing cumulative distribution function (CDF) plots shown in Fig. S8.

**Figure 4:**
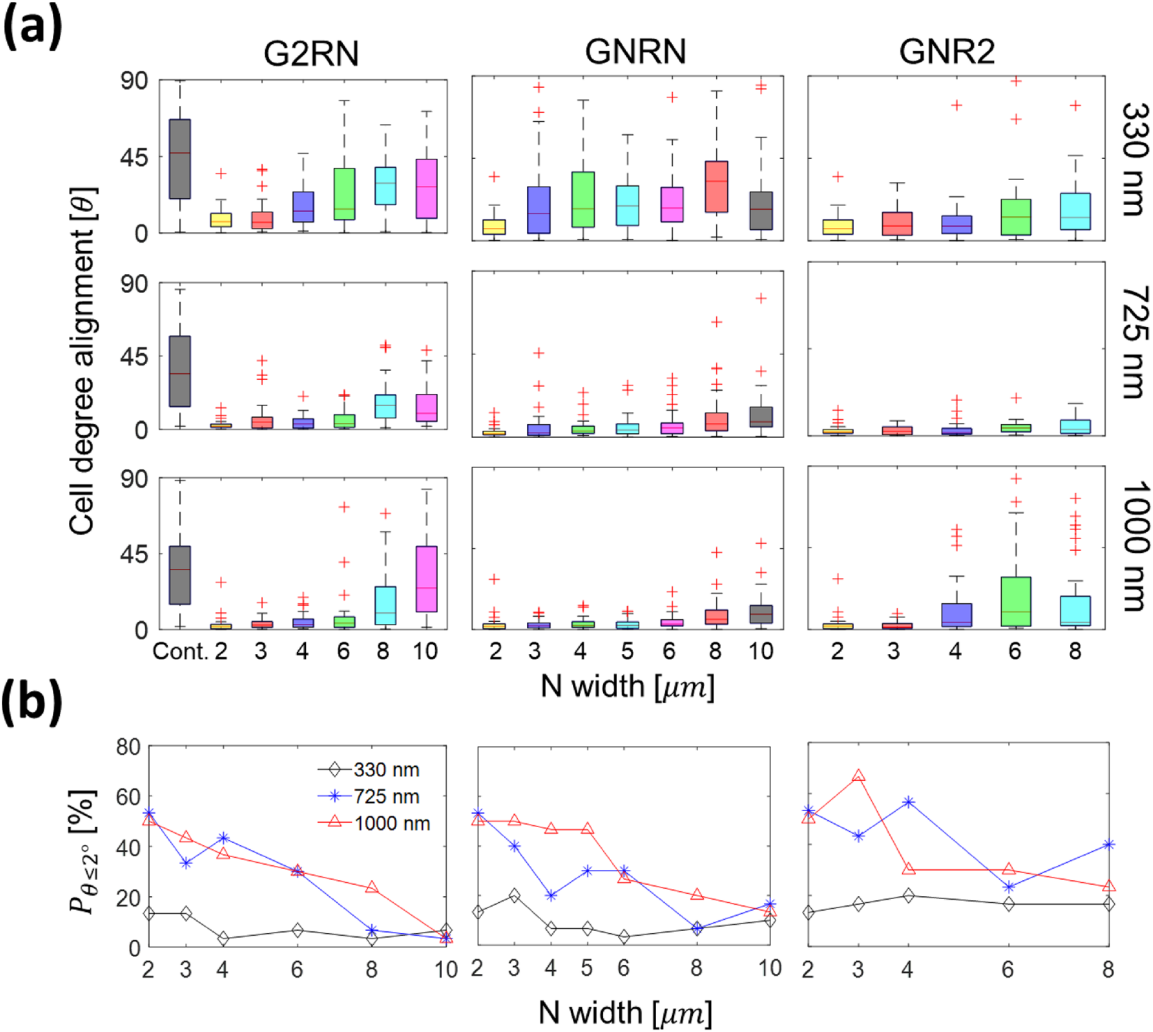
(**a**) Box-and-whisker diagrams of cell alignment angle ($\uptheta$) with respect to groove patterns. (**b**) The percentage cell population (${{P}}_{\uptheta \le 2^\circ }$) within $\theta \le 2^\circ$ under different lateral conditions (${{\text{G}}}_{{\text{w}}}$ and ${{\text{R}}}_{{\text{w}}}$).

For more detailed analyses, Fig. 4a shows the cellular alignment angle (*θ*) for G2RN, GNRN, and GNR2. Note that *θ* increases with either the increase of ${{\text{G}}}_{{\text{w}}}$ and ${{\text{R}}}_{{\text{w}}}$ or the decrease of ${{\text{G}}}_{{\text{D}}}$. For ${{\text{G}}}_{{\text{D}}}=725 {\text{ nm}}$ (the second row), the ${\theta }_{ave}$ values for G2RN, GNRN, and GNR2, increase from $2.87^\circ$ (${\text{N}}=2\,\mathrm{ \mu m}$) to $17.15^\circ$, $11.74^\circ$, and $4.69^\circ$ (${\text{N}}=8\,\mathrm{ \mu m}$), respectively. Our statistical analysis in Figs. S6 and S7 indicates that the effect of ${{\text{G}}}_{{\text{D}}}$ is more significant than ${{\text{R}}}_{{\text{w}}}$ and ${{\text{G}}}_{{\text{w}}}$ in the light of the smallest p-value caused by more statistically significant pairs, not small variance within each topography. Also, it appears that the relative influence of ${{\text{G}}}_{{\text{w}}}$ on *θ*, compared to the other dimensional cues, is the weakest by the comparison of the corresponding p-values.

Figure 4b shows the percentage population of aligned cells (${P}_{\theta \le 2^\circ }$) for different ${{\text{G}}}_{{\text{w}}}$, ${{\text{R}}}_{{\text{w}}}$, and ${{\text{G}}}_{{\text{D}}}$ values. It is worth noting that we have introduced a new criterion ($\theta \le 2^\circ$) to quantify aligned cells because of the following reasons Other prior studies^27,42,43^ categorized $\theta =$ 0–15° as aligned cells, but their standard is not adequate for our study since $>70\,\mathrm{\%}$ cells on our patterned substrates are categorized as $\theta \le 15^\circ$. On the contrary, we found that about 23% of cells on our chips satisfy $\theta \le 2^\circ$, which also corresponds to the percentage population of elongated cells in Fig. 2b. Note that our new criterion allows us to consistently analyze the trends of ${P}_{\theta \le 2^\circ }$ using the comparable size in subpopulations as ${P}_{\overline{\alpha }\ge 2}$ and, arguably, the correlation between ${P}_{\overline{\alpha }\ge 2}$ and ${P}_{\theta \le 2^\circ }$ can be considered.

For ${{\text{G}}}_{{\text{D}}}=1000 {\text{ nm}}$ (red triangle), ${P}_{\theta \le 2^\circ }$ decreases from 50% (${\text{G}}2{\text{R}}2$) to 23.3% (both ${\text{G}}2{\text{R}}8$ and G8R2), whereas the corresponding ${P}_{\theta \le 2^\circ }$ values for ${{\text{G}}}_{{\text{D}}}=330 {\text{ nm}}$ (black diamond) are 13.3% (${\text{G}}2{\text{R}}2$), 6.67% (${\text{G}}2{\text{R}}8$), and 16.7% (${\text{G}}8{\text{R}}2$). ${{\text{G}}}_{{\text{w}}}$ and ${{\text{R}}}_{{\text{w}}}$ effects are more noticeable when ${{\text{G}}}_{{\text{D}}}$ values are 725 and 1000 nm. In addition, ${P}_{\theta \le 2^\circ }$ is sensitive to ${{\text{G}}}_{{\text{D}}}$ up to ${{\text{G}}}_{{\text{D}}}=725 {\text{ nm}}$ (p < 0.001), but no statical difference between ${{\text{G}}}_{{\text{D}}}=725\,\mathrm{and}\,1000\,\mathrm{ nm}$ (p ~ 0.583) as indicated in Fig. S5b. As an example, the average ${P}_{\theta \le 2^\circ }$ values for ${{\text{G}}}_{{\text{D}}}=0,$ 330, 725, and 1000 nm, regardless of ${{\text{G}}}_{{\text{w}}}$ and ${{\text{R}}}_{{\text{w}}}$, are 3.33%, 10.6%, 29.8%, and 33.8%, respectively. It is worth noting that we performed regression analyses of ${P}_{\theta \le 2^\circ }$ using Eq. S2 and obtained similar conclusions (see Table S3 in the supplementary) compared to the regression results of the ${P}_{\alpha \ge 2}$ data. The effect of groove depth on the cell alignment is the most significant cue.

**Figure 5:**
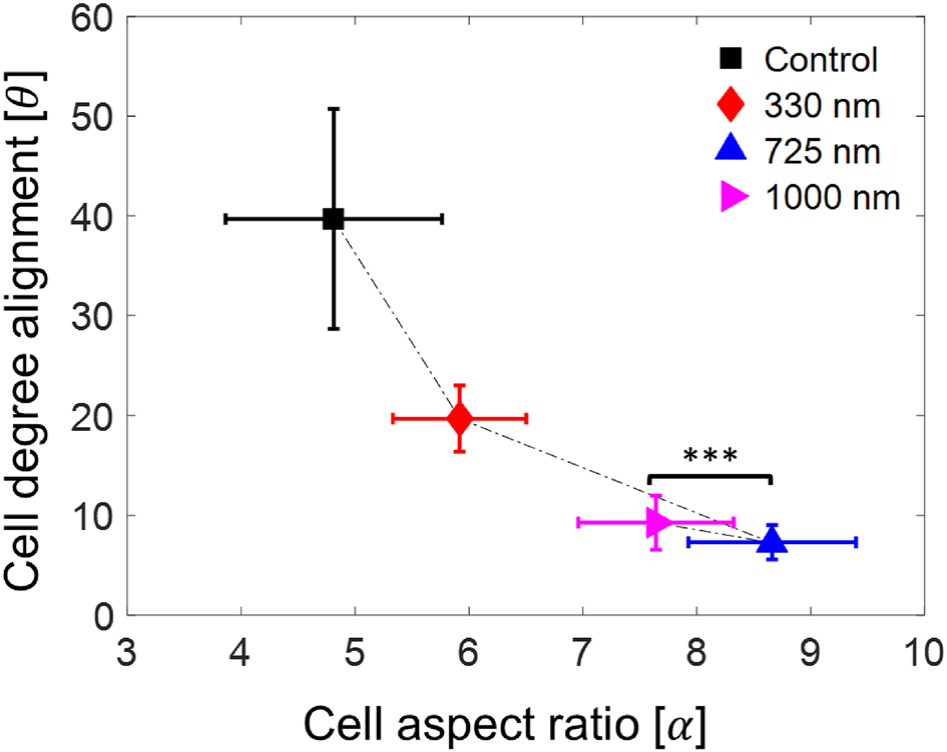
The cell alignment ($\uptheta$) as a function of the cell aspect ratio ($\mathrm{\alpha }$) for different groove depths. The error bar denotes 95% confidence level interval.

$\mathrm{\alpha }$ and $\theta$ are the most sensitive to ${{\text{G}}}_{{\text{D}}}$ among the controlled morphological cues in this study. To further consider the effect of ${{\text{G}}}_{{\text{D}}}$ on both $\mathrm{\alpha }$ and $\theta$, regardless of ${{\text{G}}}_{{\text{w}}}$ and ${{\text{R}}}_{{\text{w}}}$, Fig. 5 correlates the cell alignment angles with the corresponding cell aspect ratios for different groove depth (${{\text{G}}}_{{\text{D}}}$). Interestingly, the $\mathrm{\alpha }$–$\theta$ correlation appears to be inversely proportional. For example, for ${{\text{G}}}_{{\text{D}}}=0 ({\text{control}}), 330,\,\mathrm{and}\,725\,\mathrm{ nm}$, the trend is a decrease in $\theta$ with an increase in $\mathrm{\alpha }$. Then the direction of the trend changes from $725$ to 1000 nm as evidenced by a similar $\theta$ (p ~ 0.44), but a smaller $\alpha$ (***p < 0.001) with the considerable statistical significance.

As mentioned above, there is a significant discrepancy in the previous reported and our new $\theta$ values likely due to cell-type-dependent contact guidance. Moreover, Crouch et al.^27^ studied human foreskin fibroblasts (HFF) only using G5R5 configuration with different depths from ${{\text{G}}}_{{\text{D}}}=800\,\mathrm{ nm}$ to ${{\text{G}}}_{{\text{D}}}=1024\,\mathrm{ nm}$. Despite the nearly identical cell aspect ratio of HFF and Hs27 cells (Fig. S9) as a function of the groove depth, the corresponding cell alignment again exhibits considerable cell type dependent discrepancy. Because of these cell type dependency, time-effective characterization of cellular response to contact guidance, e.g., using multiplexed contact guidance chips to concurrently consider a wide range of topographic design parameters within the same cell culture, becomes increasingly relevant for practical optimization and application of contact guidance technologies for biomedical applications and biological studies.

### Cell migration: dynamic image analysis

In this section, we perform analyses to quantify dynamic cell migration on contact guidance topographies with an emphasis on directional migration along the groove direction. All the directional characteristics of the dynamic cell migration in the following analyses are characterized with respect to the groove direction.

**Figure 6:**
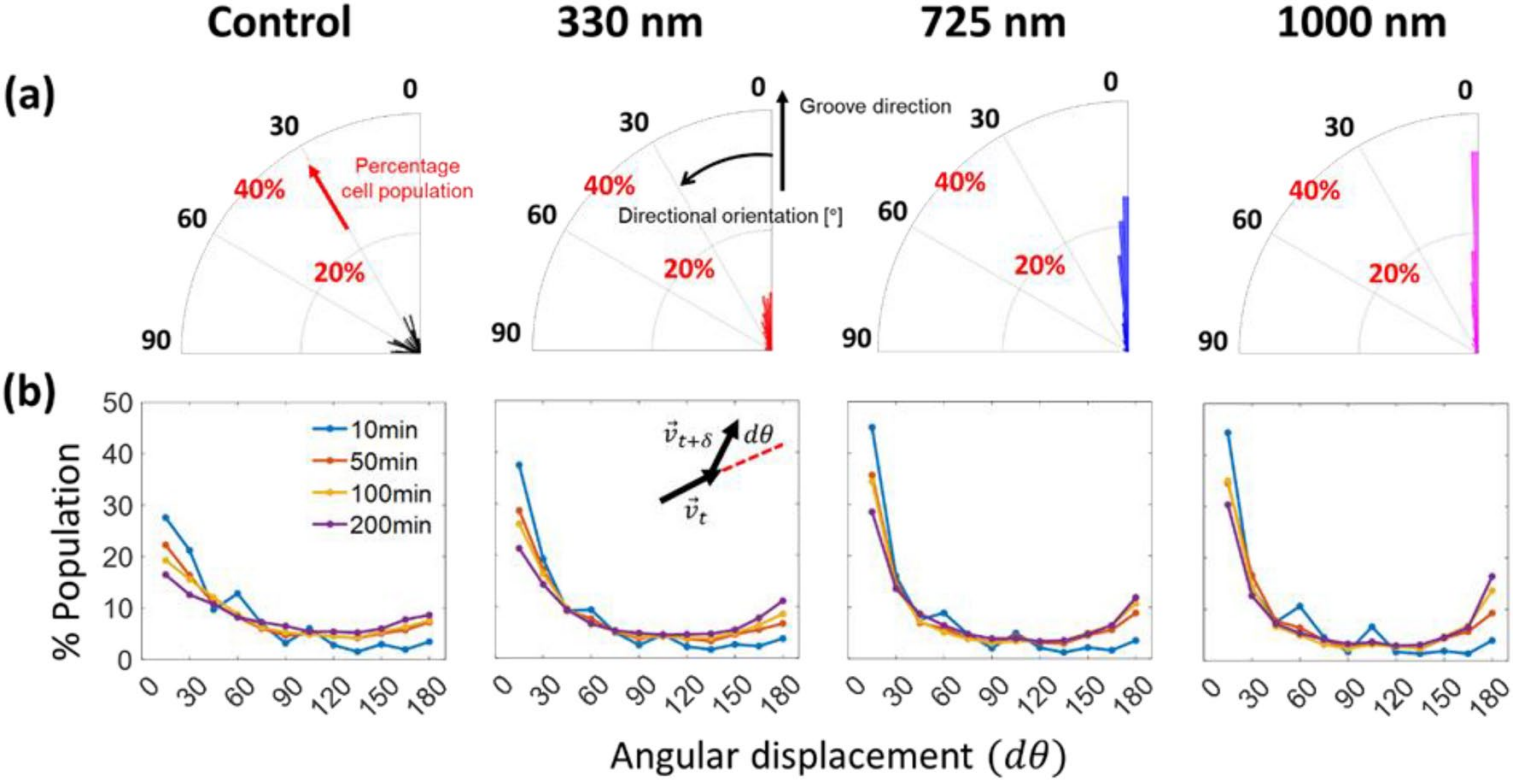
(**a**) Directional orientations of the fibroblasts on contact guidance with different groove depths. The angular and radial coordinates represent the cell orientation from $0$ to $90^\circ$ and the corresponding percentage cell population within the total population (each bin size is $2^\circ$). (**b**) The percentage population distribution of angular displacement ($d\theta$, see graphical definition in inset and $\delta$ represents a time interval) evaluated at four different time intervals (10, 50, 100, and 200 min). For (**a,b**), each column represents the results of different groove depths (left to right) from 0 to 1000 nm.

Figure 6a and Fig. S10 show the overall migration directionality. For this, vectors from the initial to final positions of the same individual cells migrational tracks > 10-h intervals were obtained from optical images. Then the angle between the vector and the groove direction was calculated for the migration directionality. Note that the percentage cell population near $0^\circ$ monotonically increases with the groove depth. For example, the cell populations between $0^\circ$ and $10^\circ$ are 8.21%, 41.7%, 71.8%, and 75.3% on ${{\text{G}}}_{{\text{D}}}=$ 0, 330, 725, and 1000 nm, respectively. For statistically significant conclusions on the ${{\text{G}}}_{{\text{D}}}$-sensitive directional orientation, we have performed Kruskal Wallis test as shown in Fig. S11a. The average directional orientations are $43.8^\circ , 21.6^\circ , 10.7^\circ ,\,\mathrm{and}\,9.83^\circ$ on ${{\text{G}}}_{{\text{D}}}=$ 0, 330, 725, and 1000 nm, respectively. The results confirm that the directional orientation decreases from ${{\text{G}}}_{{\text{D}}}=0$ to = 725 nm (${\text{p}}\ll 0.001$) and then the difference between ${{\text{G}}}_{{\text{D}}}=725$ and = 1000 nm becomes statistically insignificant (p ~ 0.06). It is worth noting that the effect of ${{\text{R}}}_{{\text{w}}}$ on the cell directional orientation is statistically obvious (p < 0.05) only for ${{\text{G}}}_{{\text{D}}}=330$ nm among three different depths (see Fig. S11b,c). Moreover, for ${{\text{G}}}_{{\text{D}}}=330 {\text{ nm}}, {{\text{R}}}_{{\text{w}}}$ has a stronger influence on the cell directional orientation than ${{\text{G}}}_{{\text{w}}}$ (i.e., ${{\text{R}}}_{{\text{w}}}:$ p ~ 0.0002 and ${{\text{G}}}_{{\text{w}}}$: p ~ 0.063), which results from more statistically significant pairs instead of small variance within each topography group as shown in Figs. S11b,c and S12.

The cell directional orientation provides the general direction of cell migration over > 10 h but misses time-dependent dynamic characteristics in a shorter period. Because of this limitation, we compute an angular displacement $(d\theta)$ of individual cells using different time intervals (i.e., $\delta =10, 50, 100$ and 200 min) and map the occurrence distribution (i.e., the normalized cell population) with a $15^\circ$-bin size using the following expression^44^:

1 $$d\theta \left(t,\delta \right)={{\text{cos}}}^{-1}([{\overrightarrow{v}}_{t}\cdot {\overrightarrow{v}}_{t+\delta }]/|{\overrightarrow{v}}_{t}||{\overrightarrow{v}}_{t+\delta }|),$$

where ${\overrightarrow{v}}_{t}$ is a velocity vector of a cell between $t$ and $t+\delta$ over a finite time interval ($\delta$) (see the inset in Fig. 6b). The occurrence in Fig. 6b exhibits two general trends for all three ${{\text{G}}}_{{\text{D}}}$ cases. First, the occurrence for $\delta =10$ min exponentially decays with increasing $d\theta$, but a larger ${{\text{G}}}_{{\text{D}}}$ value results in faster decay. Second, the occurrence in $0^\circ \le d\theta \le 15^\circ$ decreases, while the occurrence in $165^\circ \le d\theta \le 180^\circ$ increases, with increasing a time interval, respectively. These observed trends indicate that cell migration on deeper ${{\text{G}}}_{{\text{D}}}$ is indeed more directional in the groove direction for any time interval. For example, note that the occurrence in $0^\circ \le d\theta \le 15^\circ$ on ${{\text{G}}}_{{\text{D}}}=725$ and = 1000 nm (e.g., 0.285 and 0.303, respectively, for $\delta =200$ min) is significantly higher compared to ${{\text{G}}}_{{\text{D}}}=0$ nm (e.g., 0.276 even for $\delta =10$ min). In addition, the occurrence in $165^\circ \le d\theta \le 180^\circ$ for deeper ${{\text{G}}}_{{\text{D}}}$ increases with increasing $\delta$ at a faster rate compared to the control, for example, from 0.04 ($\delta =10\,\mathrm{ min}$) to 0.16 ($\delta =200\,\mathrm{ min}$) for ${{\text{G}}}_{{\text{D}}}=1000\,\mathrm{ nm}$ while the occurrence for the control remains low (i.e., 0.09 for $\delta =200\,\mathrm{ min}$). In summary, cells on deeper ${{\text{G}}}_{{\text{D}}}$ exhibit strong forward motion at shorter $\delta$ and back-and-forth motion at larger $\delta$, respectively, along the groove patterns. Our additional analysis, while not discussed in detail here, indicates that the influence of ${{\text{R}}}_{{\text{w}}}$ and ${{\text{G}}}_{{\text{w}}}$ on $d\theta$ is not significant (see Fig. S13a,b, respectively).

We next identify how individual topographical cues affect migration speed of Hs27 cells. Surprisingly, the cell migration speed difference between G2RN, GNRN, and GNR2 is insignificant and the average migration speed ($\overline{v }$ = total travel distance over total travel time) is insensitive to ${{\text{R}}}_{{\text{w}}}$ and ${{\text{G}}}_{{\text{w}}}$. Again, $\overline{v }$ strongly depends on ${{\text{G}}}_{{\text{D}}}$ (see Fig. S14). Because of this reason, we summarize cell migration speed for different groove depth, ${{\text{G}}}_{{\text{D}}}$, in Fig. S15 without separately considering ${{\text{R}}}_{{\text{w}}}$ and ${{\text{G}}}_{{\text{w}}}$. The mean value of the cell speed on the flat substrate (control) is about 0.4 $\,\mathrm{\mu m}/{\text{min}}$, which matches with the previous study using human skin fibroblasts^45^. The migration speed on the ${{\text{G}}}_{{\text{D}}}=725\,\mathrm{ nm}$ is 0.25 $\,\mathrm{\mu m}/{\text{min}}$, which is significantly lower than 0.39 and 0.35 $\,\mathrm{\mu m}/{\text{min}}$ on the control and ${{\text{G}}}_{{\text{D}}}=330\,\mathrm{ nm}$, respectively. The average cell speed on ${{\text{G}}}_{{\text{D}}}=1000\,\mathrm{ nm}$ is 0.29 $\,\mathrm{\mu m}/{\text{min}}$, slightly higher than ${{\text{G}}}_{{\text{D}}}=725$ nm. Interestingly, the observed trend in the average cell speed is qualitatively similar to the cell alignment angle in Fig. 5.

**Figure 7:**
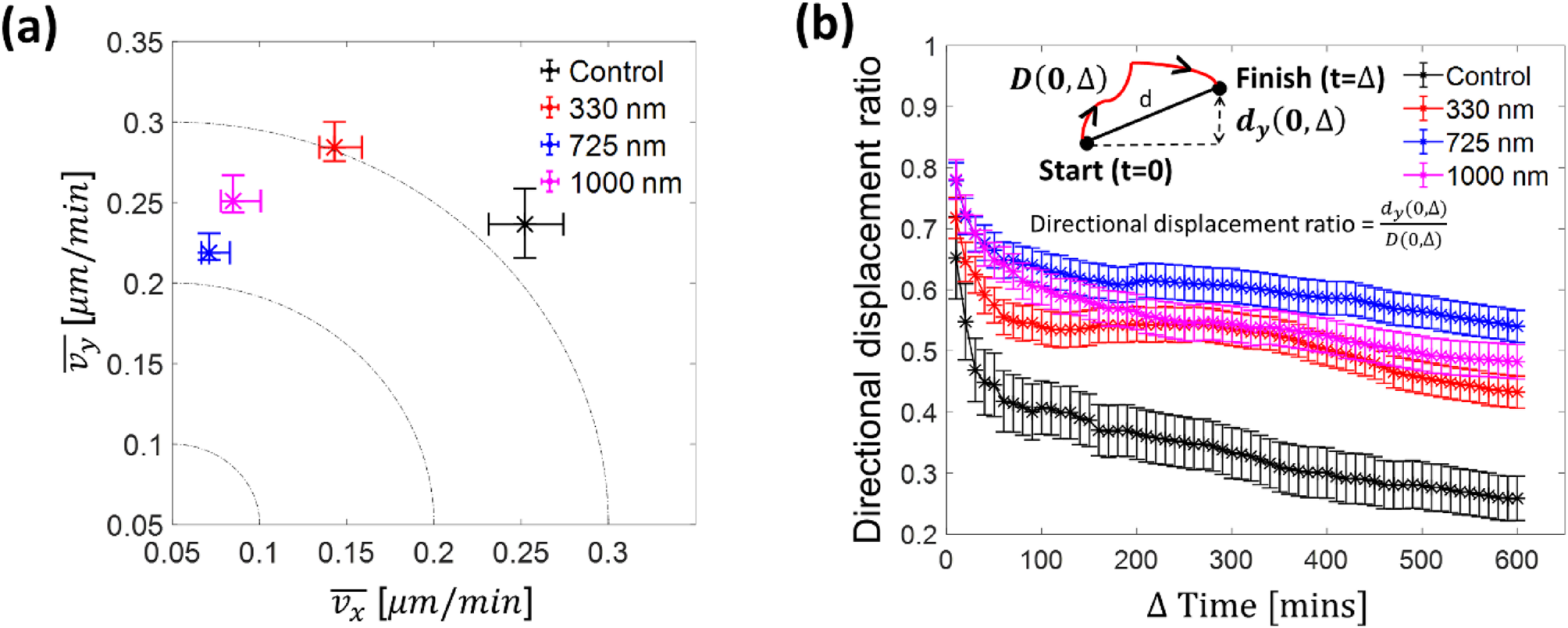
(**a**) Cell migration speed in the groove direction (${\overline{v} }_{y}$) and perpendicular to the groove direction (${\overline{v} }_{x}$). (**b**) Directional displacement ratio (i.e., y direction moving length (${{d}}_{{y}}(0,\Delta)$) divided by the total travel length ($D(0, \Delta )$) from start (*t* (time) = 0) to finish (${t}=\Delta$), see graphical definition in *inset*) as a function of the time interval ($\Delta$) for different groove depths. All error bars are mean $\pm$ standard error of mean (SEM).

To quantify the directional speed, the average migration speed is decomposed into parallel ($\overline{{v }_{y}}$) and perpendicular ($\overline{{v }_{x}}$) directions with respect to the groove direction as shown in Fig. 7a. The $\overline{{v }_{x}}$ and $\overline{{v }_{y}}$ are calculated by total travel distance along $x$ and $y$ directions over total travel time, respectively. The $\overline{{v }_{y}}$ to $\overline{{v }_{x}}$ ratio increases from 1 (control) to 3.1 (${{\text{G}}}_{{\text{D}}}=725\,\mathrm{ nm}$) with an increase of ${{\text{G}}}_{{\text{D}}}$, but it slightly decreases to 3.0 with further increases of ${{\text{G}}}_{{\text{D}}}=1000\,\mathrm{ nm}$. Note that $\overline{{v }_{y}}$ is about 3.0–3.1 times larger than $\overline{{v }_{x}}$ for ${{\text{G}}}_{{\text{D}}}=725\,\mathrm{and}\,1000\,\mathrm{ nm}$, indicating directional cell migration in the groove direction. In contrary, $\overline{{v }_{x}}\approx \overline{{v }_{y}}$ for the control experiment indicating that the cells move without a preferred direction.

So far, we have quantified the average cell speed under the influence of contact guidance. However, it is still crucial to amplify the groove direction motion (along y-axis) of cells by minimizing their lateral motion (width direction along x-axis) to effectively guide cells to target locations, e.g., injured areas, using the contact guidance technology. For an increase of migration efficiency, we consider the concept of y-axis directional persistence through a directional displacement ratio. In this section, our emphasis is placed on the effect of ${{\text{G}}}_{{\text{D}}}$ because the effect of ${{\text{G}}}_{{\text{w}}}$ and ${{\text{R}}}_{{\text{w}}}$ on cell speed is insignificant as discussed above (also see Fig. S16).

The question to be addressed is whether as an efficient directional guidance, the cell migration trajectory can be effectively controlled by a change of ${{\text{G}}}_{{\text{D}}}$ scales in order to rapidly reach our target point (i.e., somewhere along the groove direction). Similar with well-known directionality ratio (i.e., $\frac{{d}}{{D}},$ d = straight-line length between the start point and the endpoint of the migration trajectory, and *D* = the total length of the trajectory)^46^, the directional displacement ratio has been utilized to characterize y-axis directional efficiency, which is defined as follows:

2 $$\frac{{d}_{y}\left(0, \Delta \right)}{D(0, \Delta )}=\frac{y\left(\Delta \right)-y\left(0\right)}{\sum_{k=1}^{\frac{\Delta }{10}}\sqrt{{\left(x\left(10k\right)-x\left(0\right)\right)}^{2}+{\left(y\left(10k\right)-y\left(0\right)\right)}^{2}}}.$$

In other words, instead of using d, the directional displacement is the ratio of a projection of d onto the y axis (${d}_{y}\left(0, \Delta \right)$) over the total length of the trajectory ($D\left(0, \Delta \right)$) from start ($t$ (time) = 0) to finish (${t}=\Delta$) in which the time interval ($\Delta\ ({\text{min}})$) ranges from 10 to 600 min with 10 min increment as shown in the inset of Fig. 7b. Since the ${d}_{y}$ and $D$ depend on the $\Delta$, this ratio can markedly change over the time period of a cell trajectory. The directional displacement ratio for all cases in Fig. 7b decays exponentially in $\Delta$, but a larger ${{\text{G}}}_{{\text{D}}}$ value result in slower decay. More specifically, the initial values of the directional displacement ratio are 0.65 (control), 0.72 (${{\text{G}}}_{{\text{D}}}=330\,\mathrm{ nm}$), 0.78 (${{\text{G}}}_{{\text{D}}}=725\,\mathrm{ nm}$), and 0.78 (${{\text{G}}}_{{\text{D}}}=1000\,\mathrm{ nm}$) and then they decrease to 0.26, 0.43, 0.54, and 0.48, respectively, at $\Delta =600$ mins. It is worth remarking that the directional displacement ratio for the control is consistently smaller than those for all three ${{\text{G}}}_{{\text{D}}}$ cases as expected. The important observation, here, confirms that the cells require less total length of the trajectory to arrive at a target location along the groove direction with a deeper ${{\text{G}}}_{{\text{D}}}$.

## Discussion

Quantitative characterization of cell morphologies associated with a wide range of microfabricated surface patterns is relevant to understanding and guiding cell behavior for several biological applications. For example, cell spread areas have been studied in the context of cell differentiation and proliferation^47^, migration^48,49^, and transfection^50^. Note that the cell spread area becomes important for fibroblasts as it regulates cell migration in wound healing process^51^. It is also known that cell shape, often quantified by a cellular aspect ratio, is related to regulation of division-coupled interspersion for cells^39,52^. Lastly, aligned organization of cells, also known as a cell polarity, contributes to tissue maturation^53^, regeneration growth^54^, differentiation^55^, and proliferation^56^ and, as a result, a capability to control cell alignment is crucial for engineering functional tissue equivalents.

Accurate characterization of dynamic cell behavior on various topographical cues is crucial for many biological applications such as embryological development, tissue formation/regeneration, immune defense/inflammation, and cancer progression^57–59^. For instance, it is known that the directional cell movement of fibroblast enables development of a larger contact area during wound healing process^60^. Furthermore, cell migration speed is directly linked to acceleration/deceleration of pathological matrix deposition processes in fibrosis^61,62^. Finally, an overall efficiency of cell migration^63–67^, typically quantified by the directional motion and its ratio over the total travel distance, is relevant to rapid ECM synthesis and remodeling. Our study may offer a means to efficiently guide cell migration in the scope of achieving a quicker recovery of the microenvironments^68^.

In this study, we have characterized the influence of different topographical cues (i.e., ${{\text{R}}}_{{\text{w}}}, {{\text{G}}}_{{\text{w}}}, {\text{and }} {{\text{G}}}_{{\text{D}}}$) on the static and dynamic behavior of Hs27 fibroblast cells by utilizing a microfabricated contact guidance chip. To map the effect of the ${{\text{R}}}_{{\text{w}}}$ and ${{\text{G}}}_{{\text{w}}}$ beyond what has been reported in the literature, we have considered three different cases, namely, G2RN, GNRN, and GNR2. Our study on the cell morphologies shows that the cells are likely elongated and aligned with the groove direction as ${{\text{G}}}_{{\text{D}}}$ increases from 0 to 725 nm with the corresponding p-value < 10^–8^. Within the same ${{\text{G}}}_{{\text{D}}}$ range, the effect of ${{\text{R}}}_{{\text{w}}}$ is also statistically significant (p-value < 0.005). It appears that the trend in cell responses changes between ${{\text{G}}}_{{\text{D}}}=725$ and 1000 nm because the cell elongation is statistically similar while its alignment slightly decreases as evidenced by a considerable decrease in the *R*^*2*^ value.

Our experiments conclude that the groove depth, ${{\text{G}}}_{{\text{D}}}$, is the most dominant topographic cue for both static and dynamic responses of Hs27 fibroblasts, which agrees with other studies^11, 27,69^. To provide feasible explanations of the ${{\text{G}}}_{{\text{D}}}$-dependent cell behaviors, we discuss the role of cell membrane on contact guidance. When ${{\text{G}}}_{{\text{D}}}$ increases for a given ridge and groove width, cell membrane must undergo a larger degree of membrane deformation to occupy the space within the cross section of individual grooves defined by ${{\text{G}}}_{{\text{w}}} {\text{ and }} {{\text{G}}}_{{\text{D}}}$. A cell membrane protrusion led by filopodia will sense topographic cues and move toward the groove bottom. Considering that the average length of filopodia is about 1–5 μm^70–72^, the Hs27 cells will likely reach the groove bottom and form focal adhesions as confirmed in our previous study^35^. For simplicity, we assume that the lateral filopodia protrusions occur on the ridge edge with their characteristic length (${l}_{f}$). Then, the bending angle (${\theta }_{f}$) between filopodia and the groove bottom can be calculated as follows:

3 $${\theta }_{f}={{\text{sin}}}^{-1}({G}_{D}/{l}_{f}).$$

The Eq. (3) shows that ${\theta }_{f}$ increases with increasing ${{\text{G}}}_{{\text{D}}}$. In Ref.^73^, it was shown that larger ${\theta }_{f}$ increases the normal traction force component on the integrin-based filopodia adhesion. Such high normal stresses lead to an impairment of FA formation as well as severe buckling of the branched actin fibers in the filopodia^70,73,74^. As indicated by several studies^75–80^, buckled F-actin filaments shorten and break the actin shaft and, therefore, filopodia forms in the lateral direction will likely be short-lived and retracting. Moreover, Dunn et al. reported that a larger bending angle, e.g., to follow discontinuous topographic patterns on a contact guidance chip, further limits lateral protrusions of filopodia across the features^3^. Based on the previous studies, filopodia likely becomes thick, actin-rich, and stable in the groove direction compared to the lateral direction^81^. This may offer possible explanation for the directional cell orientation and migration because filopodia is known to be linked with the cell morphology^82^ as well as the cell migration^83,84^.

For the ${{\text{R}}}_{{\text{w}}}$-dependent morphology of Hs27 fibroblasts, a model based on the constrained maturation of focal adhesions (FAs)^16,85^ can provide mechanistic insights. The anisotropic and oriented topographical cues, i.e., parallel ridges and grooves, likely induce cell membrane protrusions as well as FA formation and maturation along the topography. These laterally restricted FAs result in the formation of aligned actin stress fibers to along the parallel direction of the topography because the orientation of the stress fibers follows that of the FAs^35^. Note that the aligned actin stress fibers connected to large, long-lived, and oriented FAs are longer and more stable than small, short-lived, and non-aligned ones associated with non-oriented FAs^16^. With reduction of ${{\text{R}}}_{{\text{w}}}$, the formation of FAs is more confined along the groove direction and, as a result, the Hs27 fibroblasts tend to be more elongated and aligned in the same direction as shown in Fig. 4.

Our dynamic analyses also confirm ${{\text{G}}}_{{\text{D}}}$ as the main geometrical parameter to influence cell behavior responses. The cells on deeper ${{\text{G}}}_{{\text{D}}}$ are better guided along the groove direction. Cells on the flat control and ${{\text{G}}}_{{\text{D}}}=330 {\text{ nm}}$ substrates move faster, compared to those on ${{\text{G}}}_{{\text{D}}}=725 \,\mathrm{and\, }1000\,\mathrm{ nm}$. This trend ($\overline{v }=0.55\,\mathrm{ \mu m}/{\text{min}}$ for control and $\overline{v }=0.36\,\mathrm{ \mu m}/{\text{min}}$ for the grooved pattern) has also been observed by other study which uses NIH 3T3 fibroblasts with ${{\text{R}}}_{{\text{w}}}={{\text{G}}}_{{\text{w}}}=0.43\,\mathrm{ \mu m} {\text{ and }} {{\text{G}}}_{{\text{D}}}=200\,\mathrm{ nm}$^86^. However, these results are directly opposite with those of another study which reports that faster migration speed for MC3T3-E1 mouse osteoblast cells on various ${{\text{G}}}_{{\text{D}}}$ ranging from 0 ($\overline{v }=0.39\,\mathrm{ \mu m}/{\text{min}}$) to 4.5 $\,\mathrm{\mu m}$ ($\overline{v }=0.61\,\,\mathrm{ \mu m}/{\text{min}}$) with ${{\text{R}}}_{{\text{w}}}=6\,\mathrm{ \mu m}$ and ${{\text{G}}}_{{\text{w}}}=4\,\mathrm{ \mu m}$^20^. Based on a positive feedback loop between actin flow rates and the cell elongation, the elongated cells are likely to accelerate their actin retrograde flow such that it improves their motility as confirmed using several different cell types such as retinal pigment epithelial cells and bone marrow-derived mice dendritic cells^15^. This discrepancy may come from not only cell type-, but substrate topography dependent characteristics.

## Conclusion

With growing interests in the development of the next generation biomaterials, a fundamental understanding of contact guidance offers the potential to guide specific cellular functions in vivo. Our current work characterizes static and dynamic behaviors of the Hs27 fibroblasts in the wide range of groove and ridge dimensions and offers better understanding of individual geometric factor’s contribution on the cellular responses. For static results, groove depth (${{\text{G}}}_{{\text{D}}}$)﻿ as deep as 725 nm induces significant elongation (${\mathrm{\alpha }}_{{\text{ave}}}=8.66$) and alignment (${\theta }_{{\text{ave}}}=7.23^\circ$), while further increase of﻿﻿ ${{\text{G}}}_{{\text{D}}}$ to 1000 nm slightly decreases both elongation (${\mathrm{\alpha }}_{{\text{ave}}}=7.64$) and alignment (${\theta }_{{\text{ave}}}=9.27^\circ$) compared to the cells on a flat substrate (control, ${\mathrm{\alpha }}_{{\text{ave}}}=4.81$ and ${\theta }_{{\text{ave}}}=39.7^\circ$). The regression analyses show that ${{\text{G}}}_{{\text{D}}}$ and ${{\text{G}}}_{{\text{D}}}\cdot {{\text{R}}}_{{\text{w}}}$ terms have a statistically significant effect on the cell elongation and alignment, whereas the effect of ${{\text{G}}}_{{\text{D}}}$ as deep as 1000 nm has been diminished compared to that of ${{\text{G}}}_{{\text{D}}}=725 {\text{ nm}}$ based on its coefficient reduction. In our dynamic analysis, a deeper ${{\text{G}}}_{{\text{D}}}$ better guided the cells migrating along the groove direction, even though their average speed slightly decreases from 0.39 $\,\mathrm{\mu m}/{\text{min}}$ (control) to 0.29 $\,\mathrm{\mu m}/{\text{min}}$ (1000 nm), respectively. The high occurrence of the $0^\circ \le d\theta (\,\mathrm{angular \,displacement})\le 15^\circ$ and $165^\circ \le d\theta \le 180^\circ$ from the groove direction and its faster decaying rate with increasing $d\theta$ indicate the y-axis directional movement of the cells on the deeper ${{\text{G}}}_{{\text{D}}}$. The large ratio of directional speed and -directional displacement, both in the groove direction, for a deeper ${{\text{G}}}_{{\text{D}}}$ suggest that the cells on the deeper ${{\text{G}}}_{{\text{D}}}$ are better guided by minimizing their unwanted motion perpendicular to the groove direction. These findings demonstrate the importance of ${{\text{G}}}_{{\text{D}}}$ for the Hs27 fibroblasts as a guideline of the efficient migration journey. Our in-depth experimental and statistical studies would provide a way of creating an effective platform for the application of various topographical cues, which are shown to be successful in guiding and controlling cell morphological and migrating characteristics.

Our multiplexed, unidirectional groove structures enable us to systematically investigate the effect of each physical constraint on the cellular responses with tightly controlled other substrate features including surface chemistry and mechanical properties (e.g., stiffness). The microfabricated topographical features aim at mimicking physiological topography such as aligned fibers and ECM tracks that cells frequently encounter in vivo. With the use of our topography, we can design well-controlled and simplified in vitro systems that allow us to tightly control cell static and dynamic behaviors as close as those on the natural surroundings. This attractive feature may offer unique research opportunities to simulate cell morphology dependent behaviors, e.g., how cell membrane damage is linked to cell morphology.

### Supplementary Information

Supplementary Information.

## Data Availability

The datasets used and/or analyzed during the current study are available from the corresponding author on reasonable request.
