# Supplementary material for: Hs27 fibroblast response to contact guidance cues
Source: Sci Rep. 2023 Dec 7;13:21691. doi: 10.1038/s41598-023-48913-9 (PMC10709656; doi:10.1038/s41598-023-48913-9)
Supplement: Supplementary file 1 — Supplementary Information. [file 41598_2023_48913_MOESM1_ESM.docx]

**Supplementary for Hs27 Fibroblast Response to Contact Guidance Cues**

C. Kim^1^, M. Robitaille^2^, J. Christodoulides^2^, Y. Ng^1^, M. Raphael^2^, and W. Kang^1,^*

^1^Arizona State University

^2^US Naval Research Laboratory

*: [wonmo.kang@asu.edu](mailto:wonmo.kang@asu.edu)

| Groove depth ($nm$) | Ridge, R ($\mu m)$ | Groove, G ($\mu m)$ |
| --- | --- | --- |
| 330  725  1000 | 2 | 2 |
|  | 2 | 3 |
|  | 2 | 4 |
|  | 2 | 6 |
|  | 2 | 8 |
|  | 2 | 10 |
|  | 3 | 2 |
|  | 3 | 3 |
|  | 4 | 2 |
|  | 4 | 4 |
|  | 5 | 5 |
|  | 6 | 2 |
|  | 6 | 6 |
|  | 8 | 2 |
|  | 8 | 8 |
|  | 10 | 10 |

Table S1. Dimensions of nano- and micro features on the substrate surfaces

Figure S1. An original optical image of live Hs27 cells on $G_{w} \left( width of groove \right)=R_{w}\left( width of ridge \right)=10\mu m with G_{D} (groove depth)=330 nm$ (left). The processed image of the left original image with the outline of each cell (right). The images in the second row show the 10x phase contrast image (left image in the second row) and the corresponding phalloidin stained image (right image in the second row) for the F-actin of the fibroblast on G10R10 with 1000 nm groove depth.


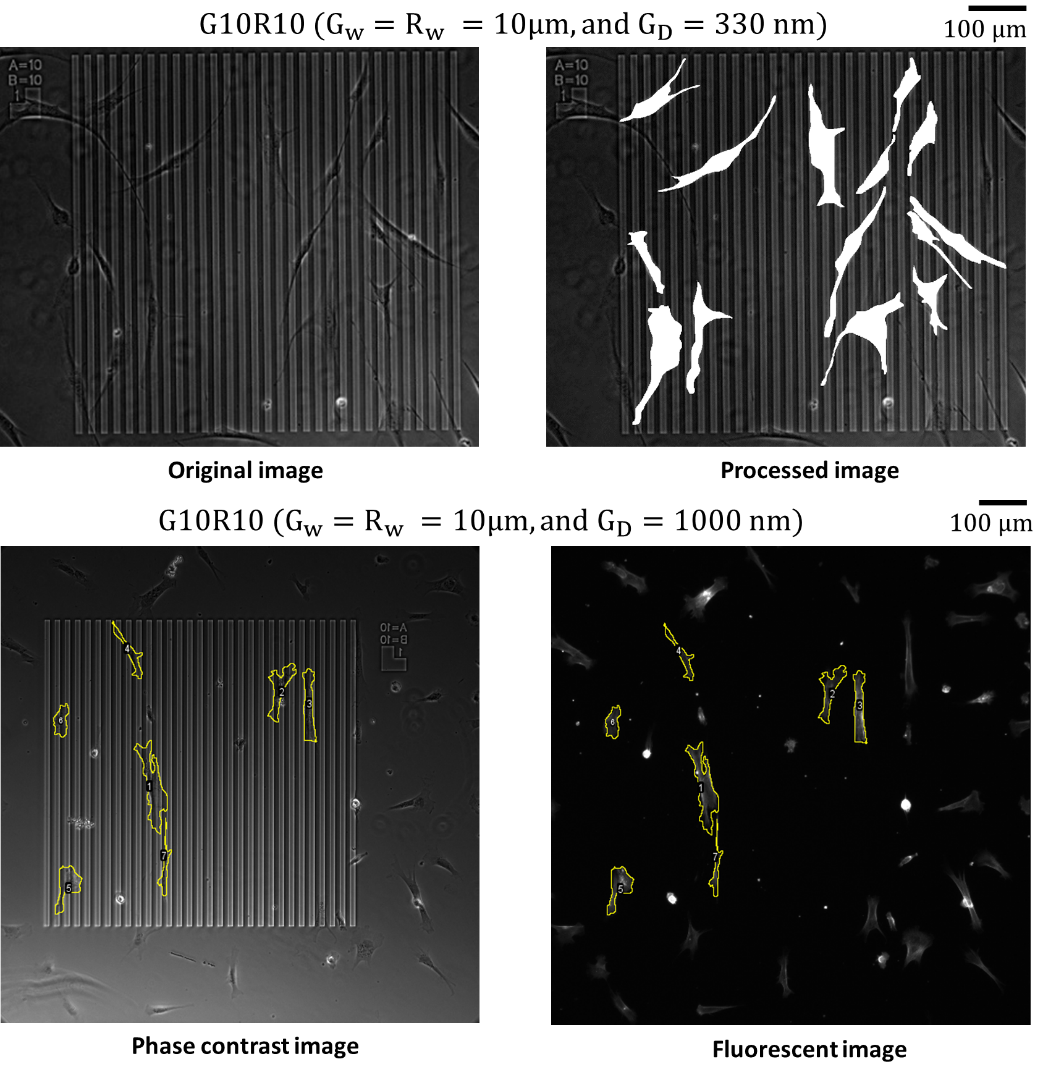

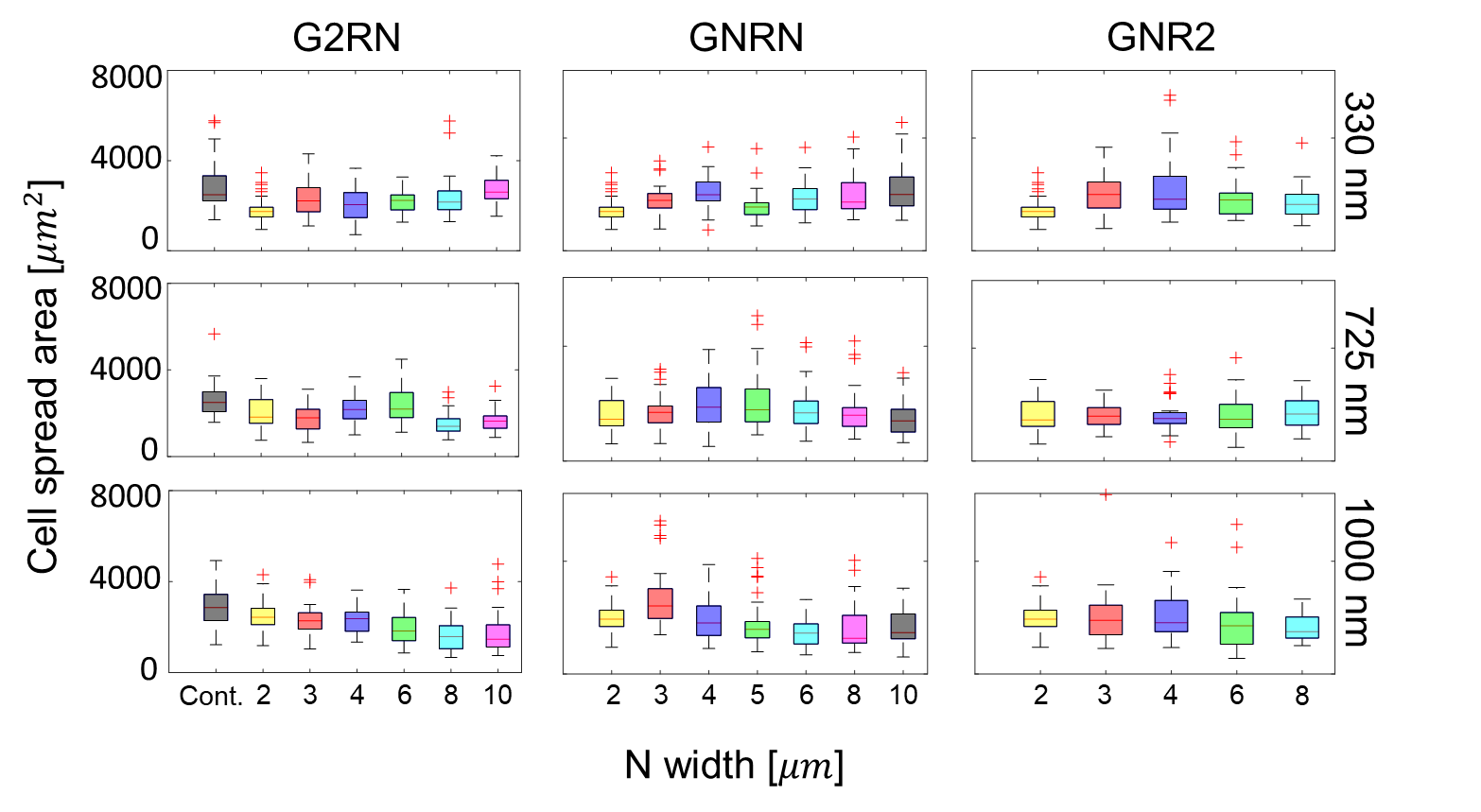


Figure S2. Box-and-whisker diagrams of cell spread area with respect to individual topographic conditions. Each column represents three different cases: (from left to right) G2RN ($G_{w}=2 \mu m$ and $R_{w}=N \mu m$), GNRN ($G_{w}=R_{w}=N$), and GNR2 ($G_{w}=N \mu m$ and $R_{w}=2 \mu m$). Each row indicates different groove depths: (from top to bottom) $G_{D}=$330, 725, and 1000 nm. Cont. indicates a control experiment on a flat control substrate.

Figure S3. Box-and-whisker plot of the cell aspect ratio as a function of groove widths (a), ridge widths (b), and groove depths (c) with the corresponding p-value from Kruskal Wallis test. *p < 0.05, **p < 0.005, and ***p<0.001 via Dunn-Sidak post hoc test.


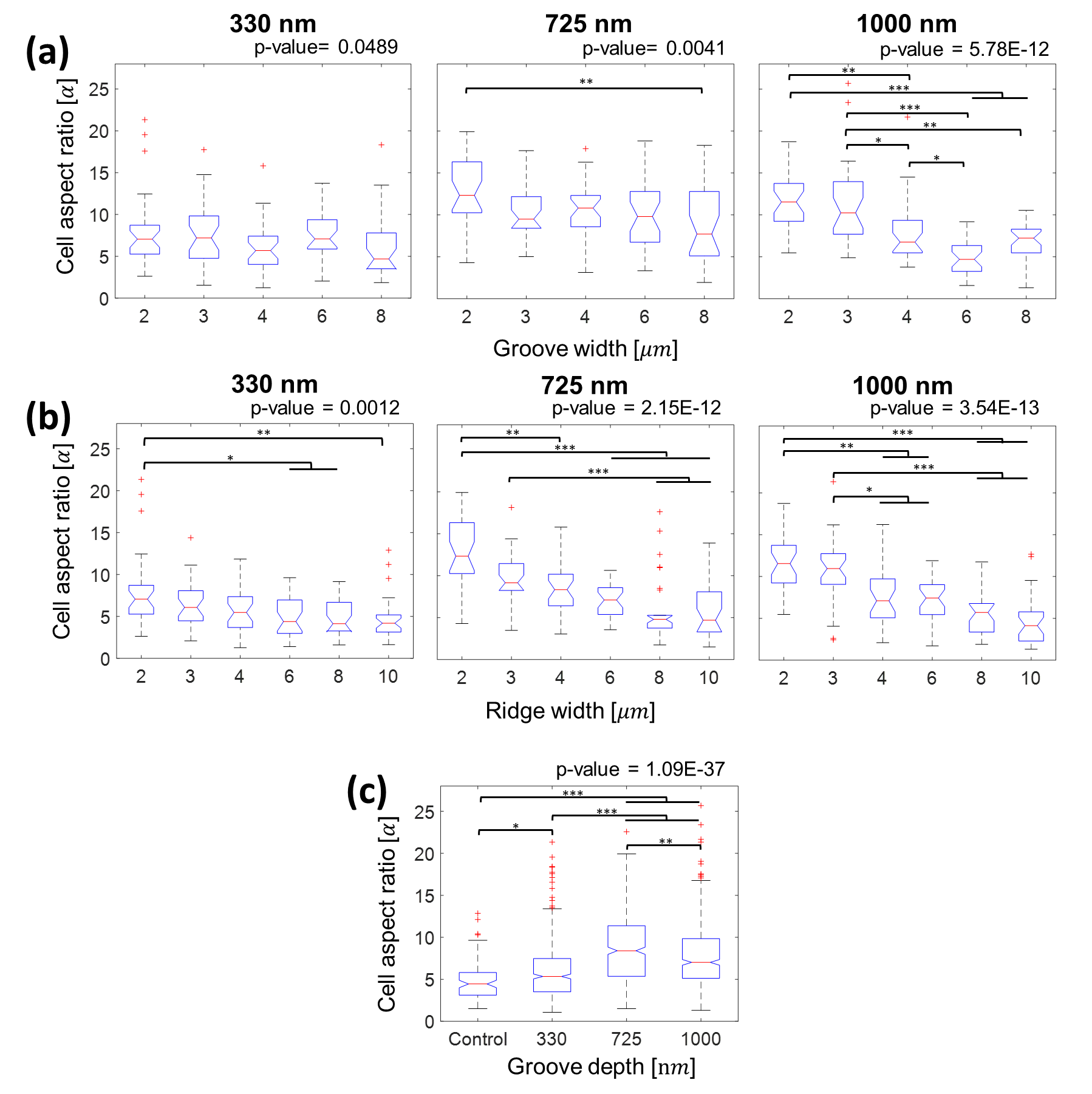

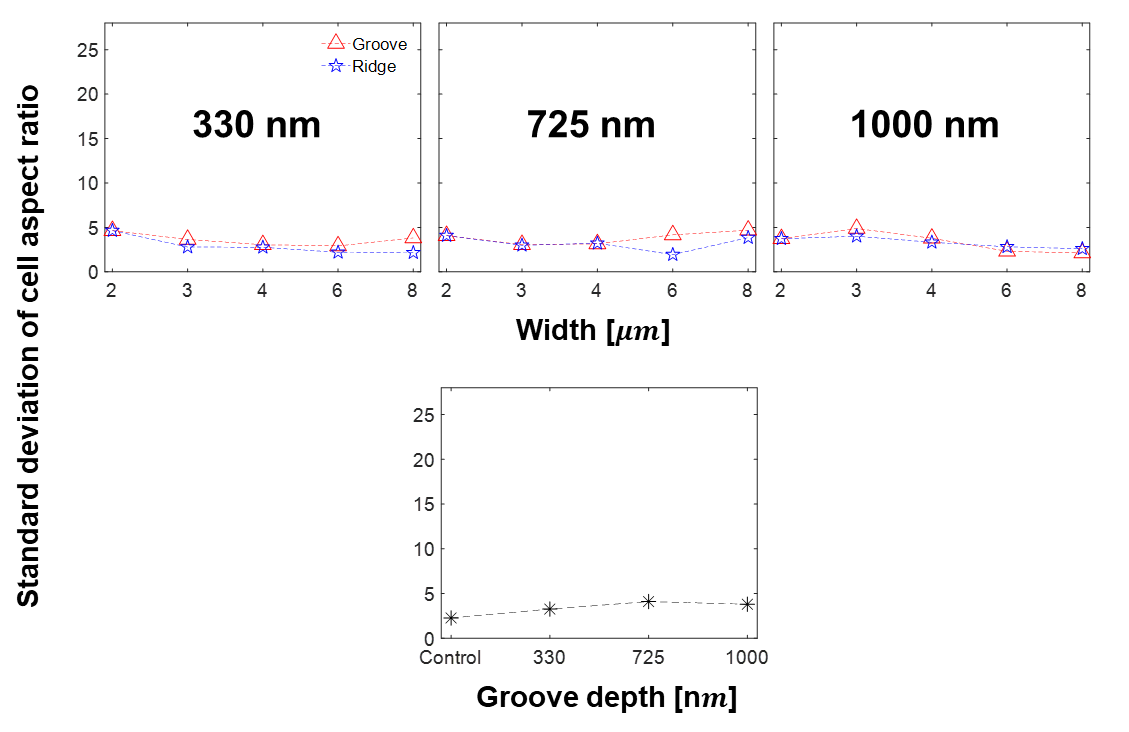


Figure S4. Standard deviation of cell aspect ratio for varying ridge widths and groove widths with different groove depths (first row) and varying groove depths (second row).

Figure S5. Box-and-whisker plot of the percentage population of the elongated, $\alpha\geq2$, (a) and aligned, $\theta\geq2^{\circ},$ cells (b) as a function of groove depths (330, 725, and 1000 nm). *p < 0.05 and ***p < 0.001 via Dunn-Sidak post hoc test.


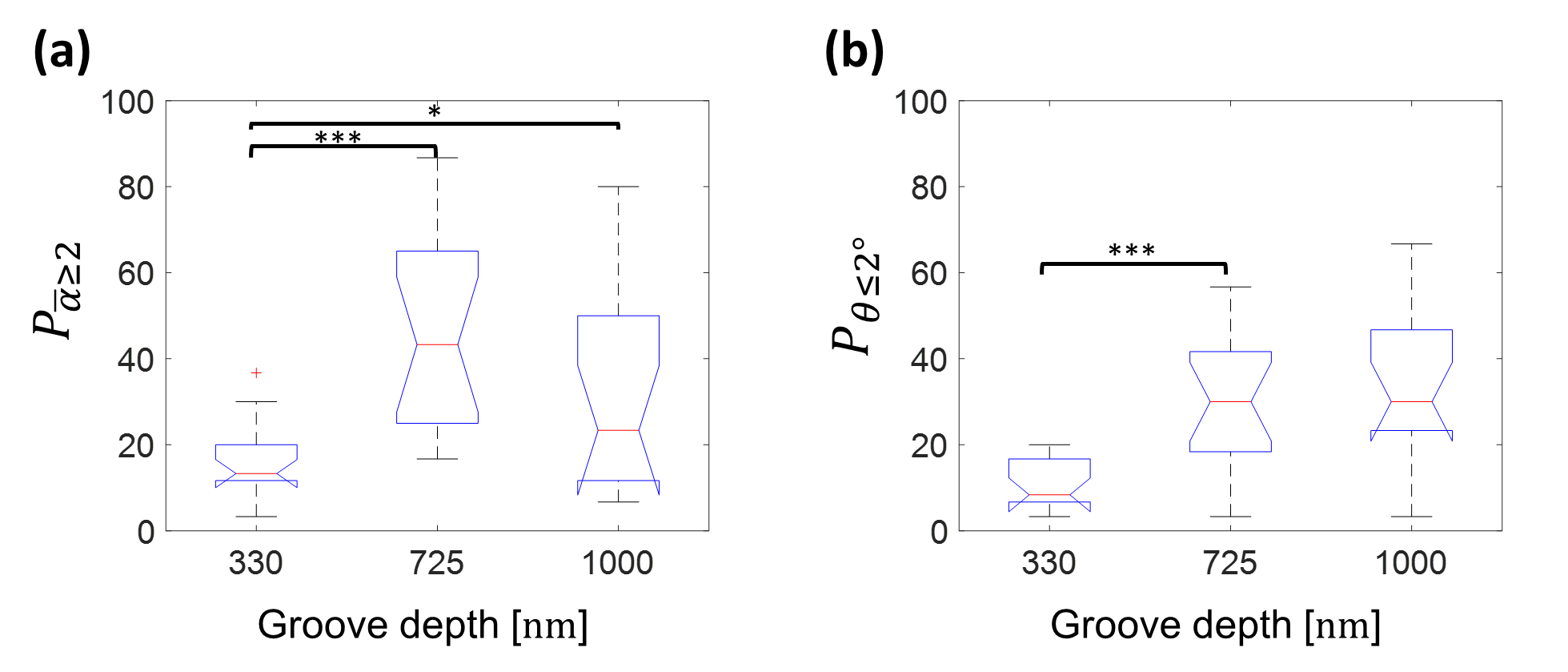

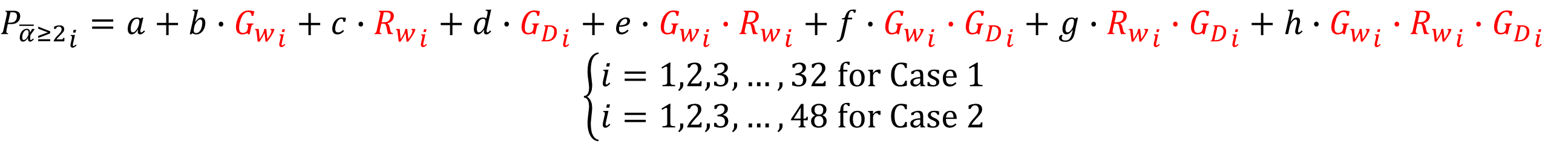


Eq S1. The multiple linear regression model of normalized elongated cell population ($P_{\bar{a}\geq2}$) for Case 1 (Control, 330, and 725 nm) and Case 2 (Control, 330, 725, and 1000 nm).

Table S2. Regression results of normalized elongated cell population ($P_{\bar{\alpha}\geq2}$) with respect to $G_{w}, R_{w}, G_{D}$, and their cross terms. Frist and second represent estimated coefficients and their p-value with $R^{2}$ value for case 1 (Control, 330, and 725 nm) and case 2 (Control, 330, 725, and 1000 nm), respectively.


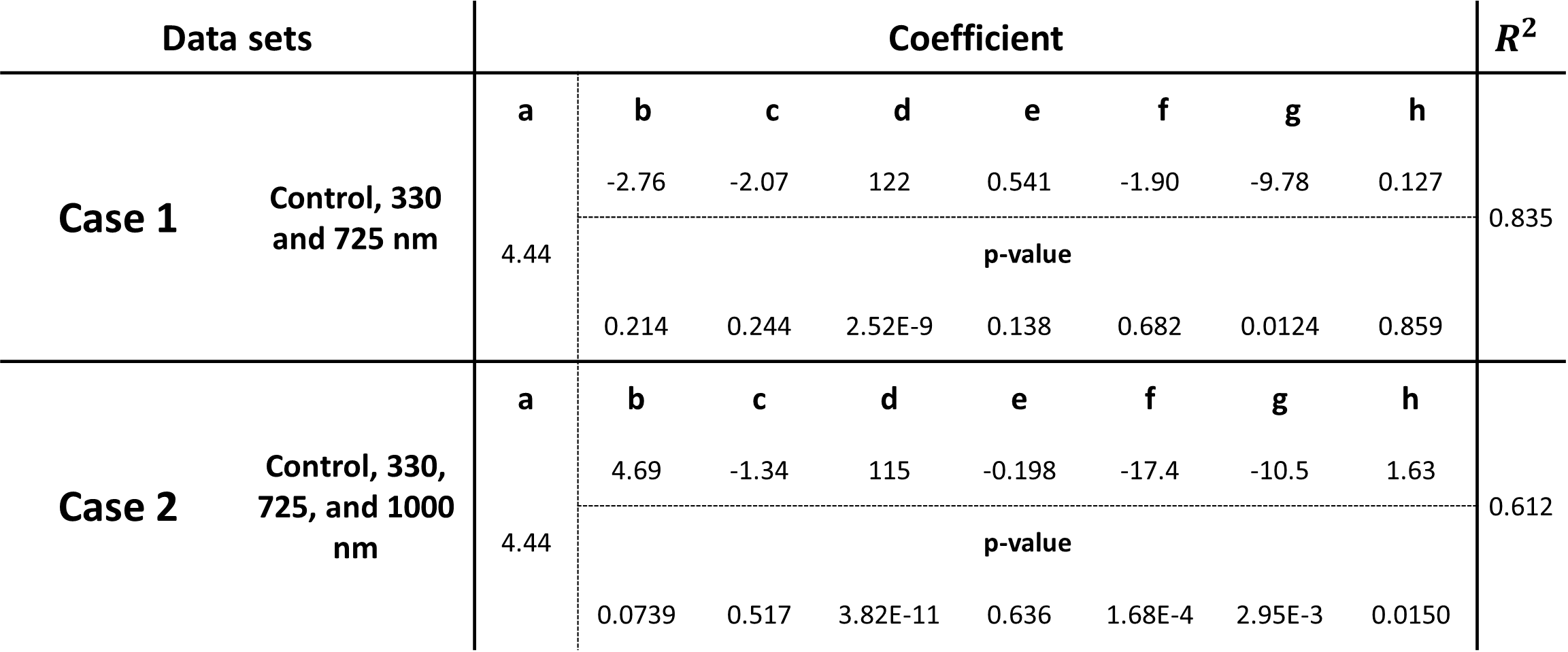


Figure S6. Box-and-whisker plot of the cell degree alignment as a function of groove widths (a), ridge widths (b), and groove depths (c) with the corresponding p-value from Kruskal Wallis test. *p < 0.05, **p < 0.005, and ***p<0.001 via Dunn-Sidak post hoc test.


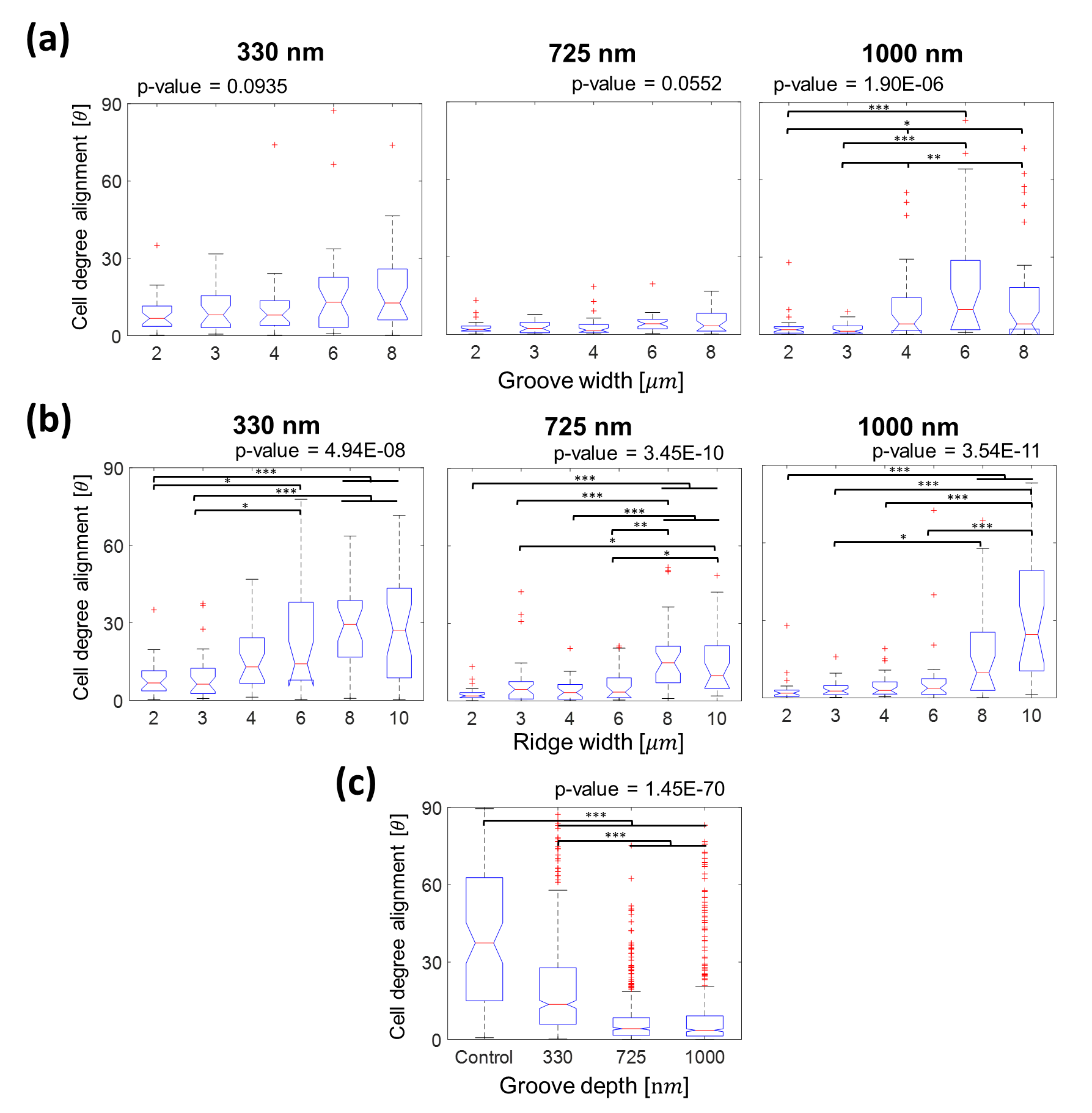

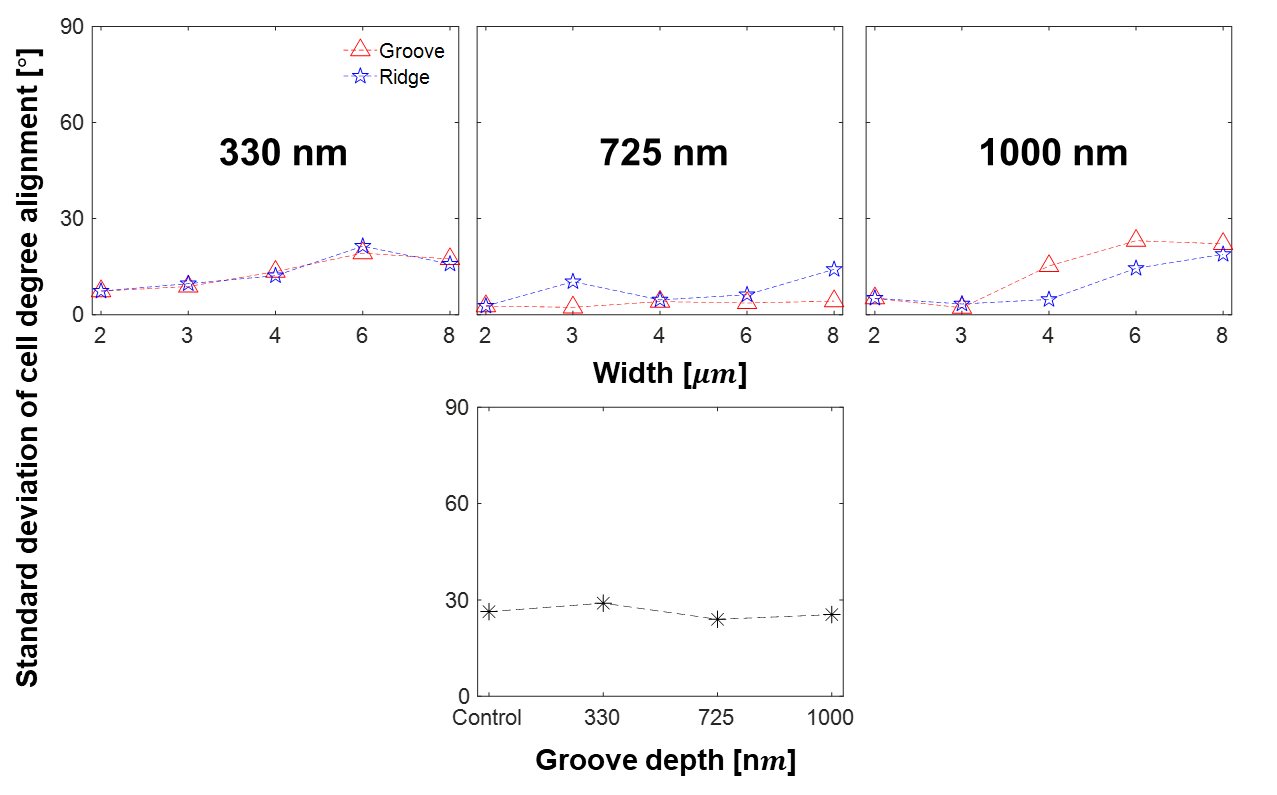


Figure S7. Standard deviation of cell degree alignment for varying ridge widths and groove widths with different groove depths (first row) and varying groove depths (second row).


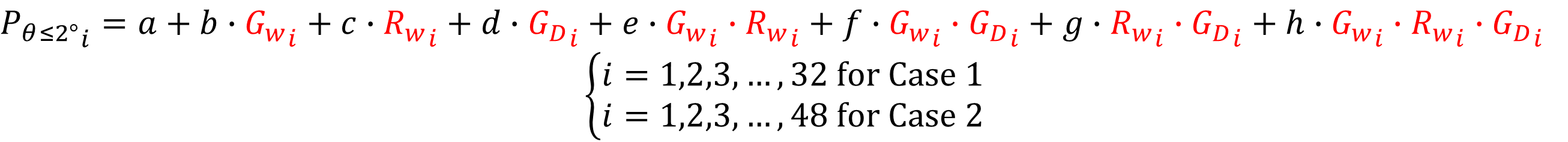


Eq S2. The multiple linear regression model of normalized aligned cell population ($P_{\theta\leq2^{\circ}}$) for Case 1 (Control, 330, and 725 nm) and Case 2 (Control, 330, 725, and 1000 nm).


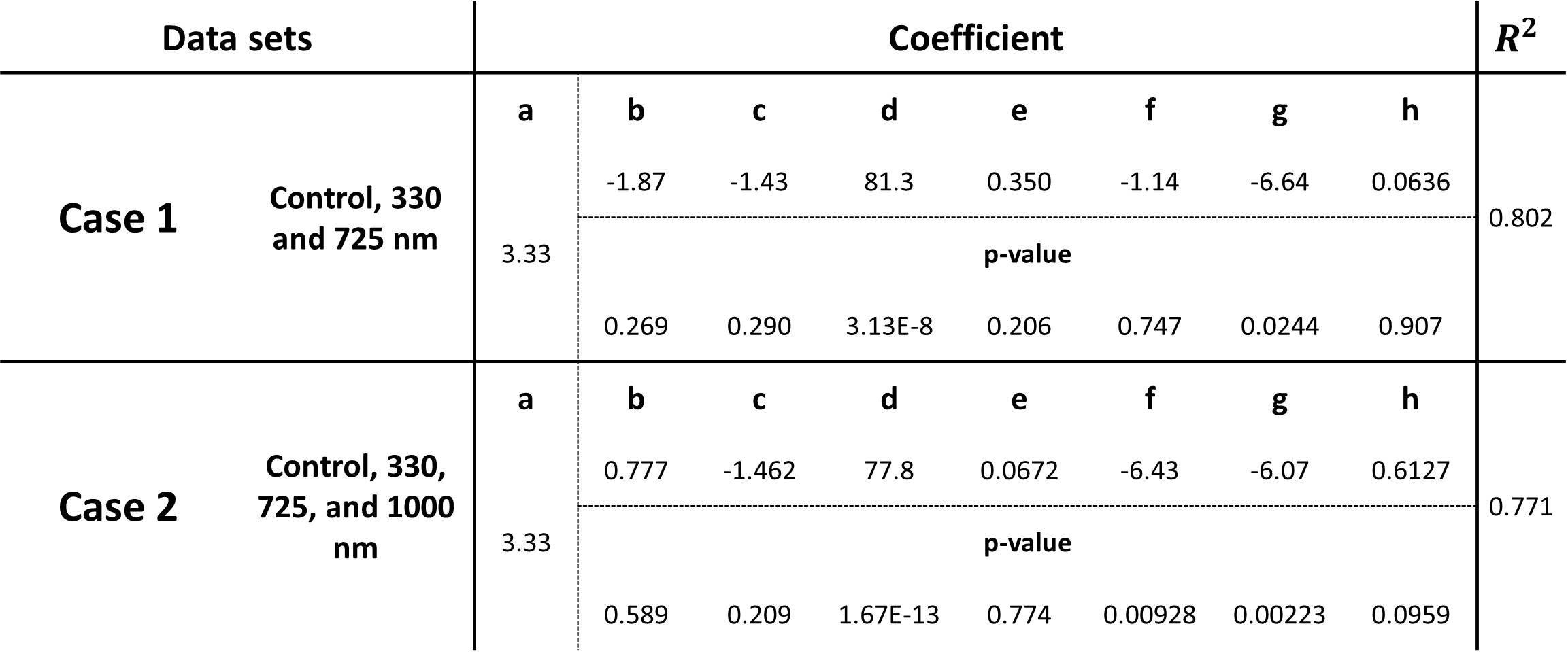


Table S3. Regression results of normalized aligned cell population ($P_{\theta\leq2^{\circ}}$) with respect to $G_{w}, R_{w}, G_{D}$, and their cross terms. Frist and second represent estimated coefficients and their p-value with $R^{2}$ value for case 1 (Control, 330, and 725 nm) and case 2 (Control, 330, 725, and 1000 nm), respectively.


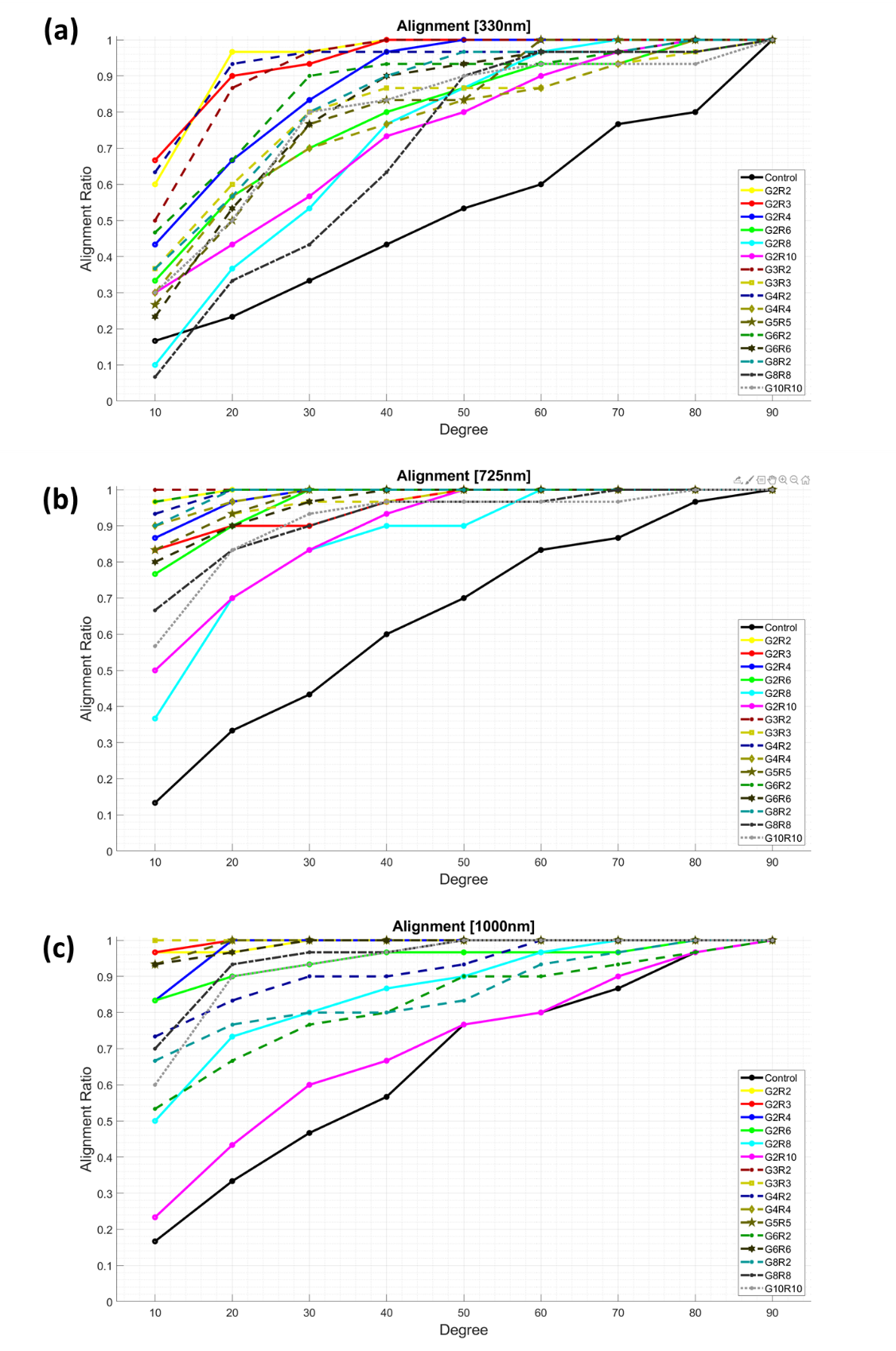


Figure S8. Graphs of the radial distribution of the major axes of Hs27 cells on various topographies. Distribution of ratio of aligned cells within a specific degree. Results for each substrate are labelled with different colors and line types ((a): 330 nm depth, (b): 725 nm depth, and (c): 1000 nm depth).


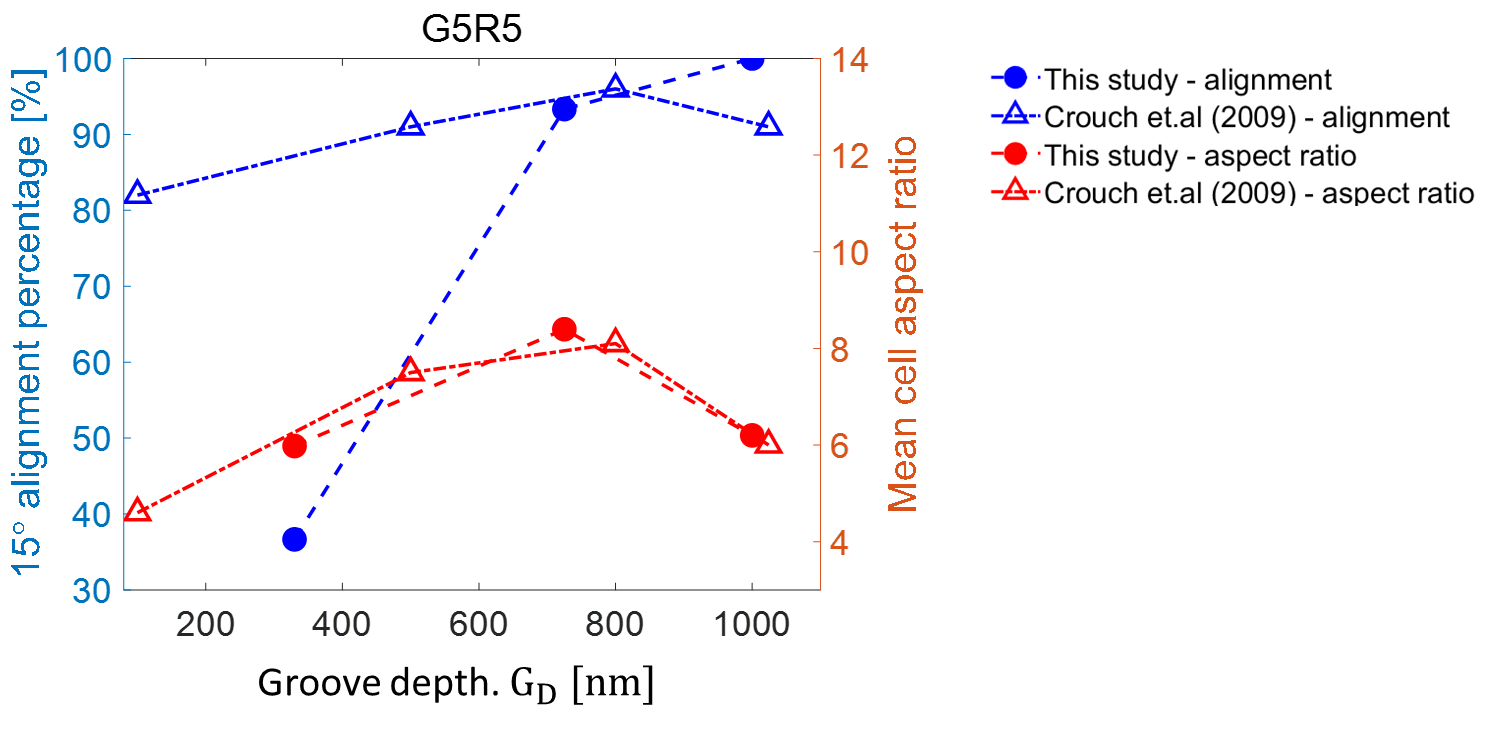


Figure S9. A plot for the $15^{\circ}$ alignment percentage (blue) and mean cell aspect ratio (red). The solid circle represents the results of our study, while the empty triangular indicates the study from Crouch et al (2009).

Figure S10. Directional orientations for the fibroblasts on all topographical cues in a polar plot. The range of the phase angle is from $0$ to $90^{\circ}$ (the bin is $2^{\circ}$). The radial coordinate represents the normalized number of cells having a directional angle within the phase angle intervals. The major grid intervals for phase and radial coordinates indicate $30^{\circ}$ and $0.125$, respectively.


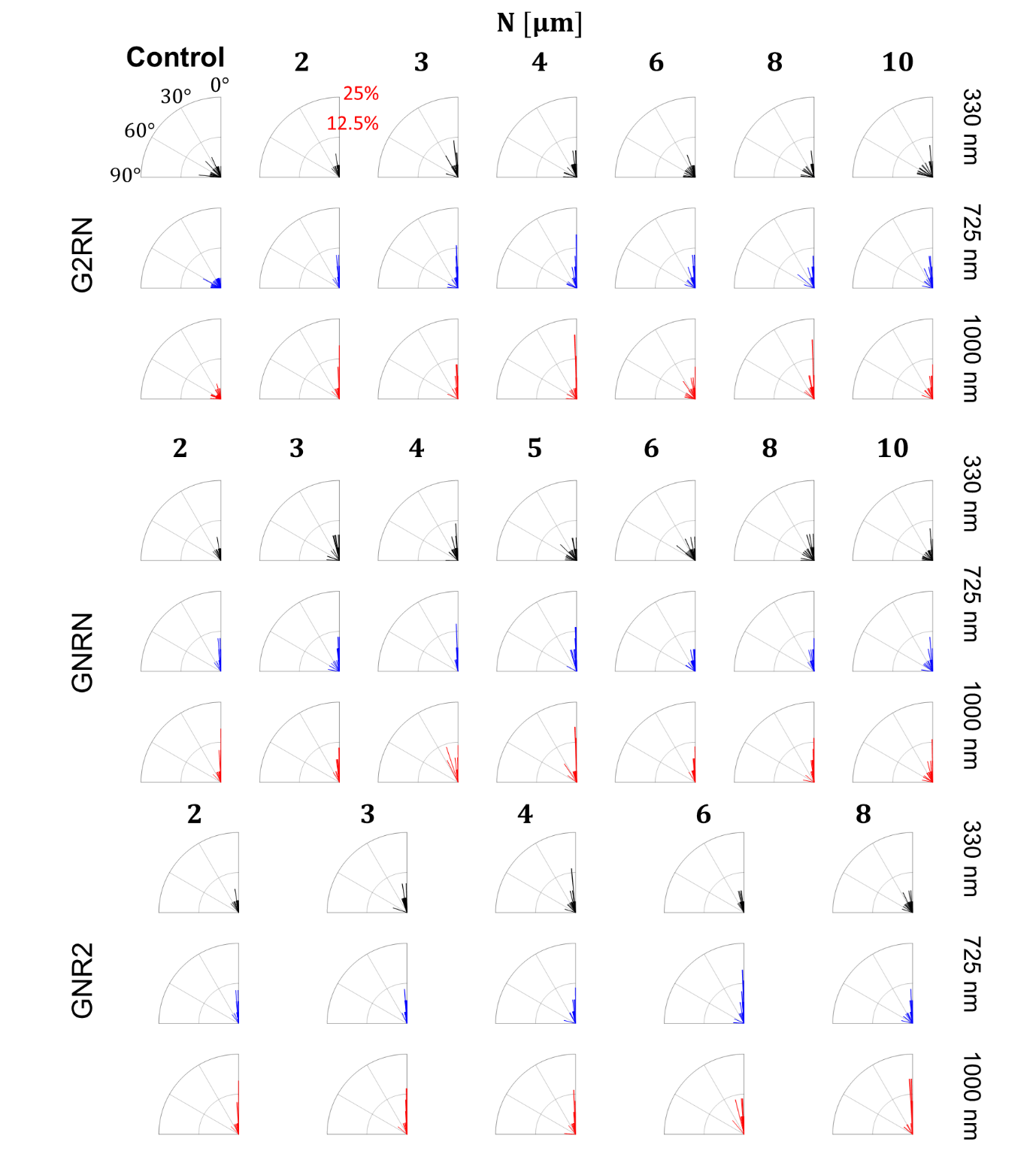


Figure S11. Box-and-whisker plot of the directional orientation as a function of groove depths (a), ridge widths (b), and groove widths (c) with the corresponding p-value from Kruskal Wallis test. *p < 0.05 and ***p<0.001 via Dunn-Sidak post hoc test.


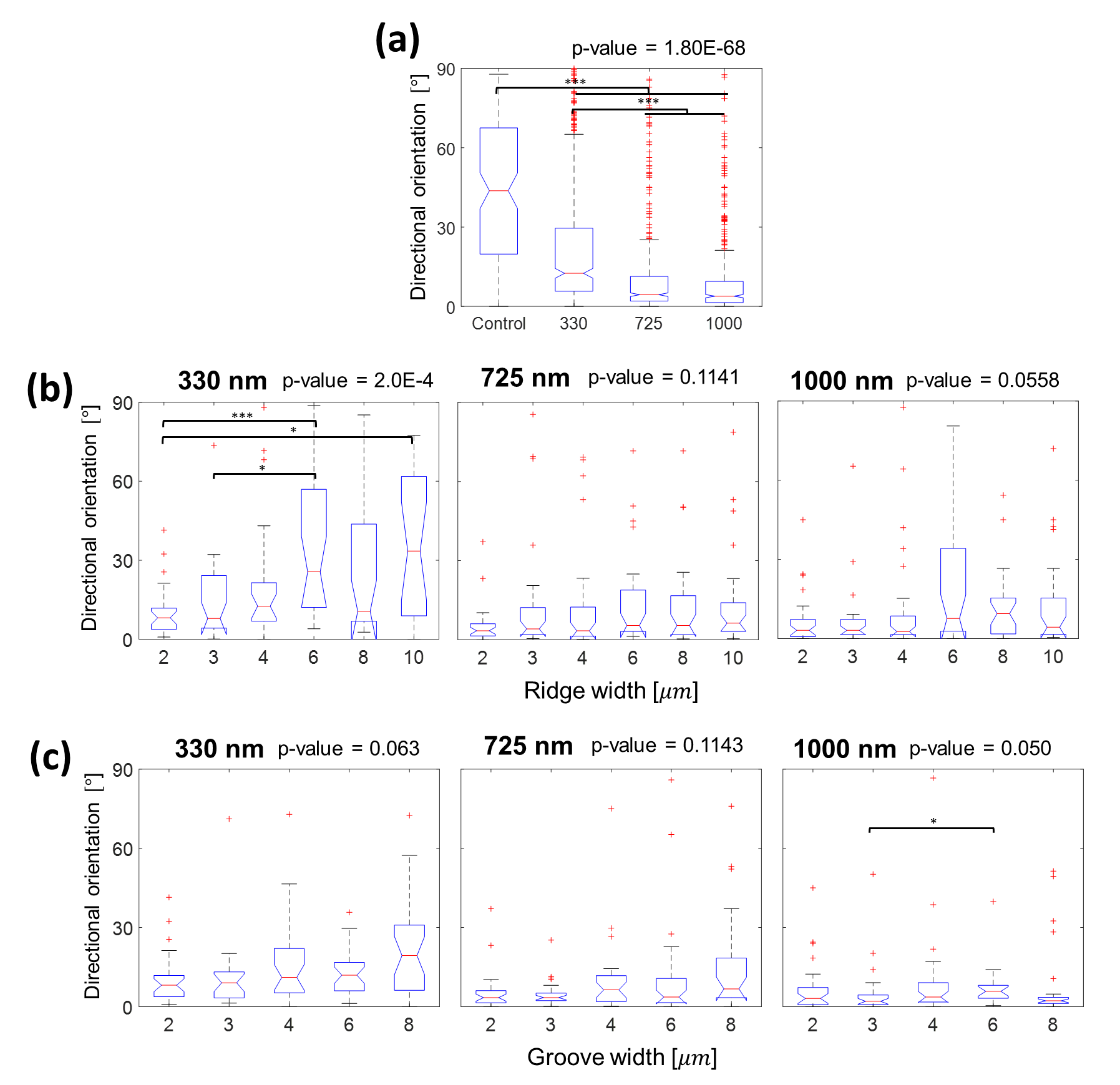

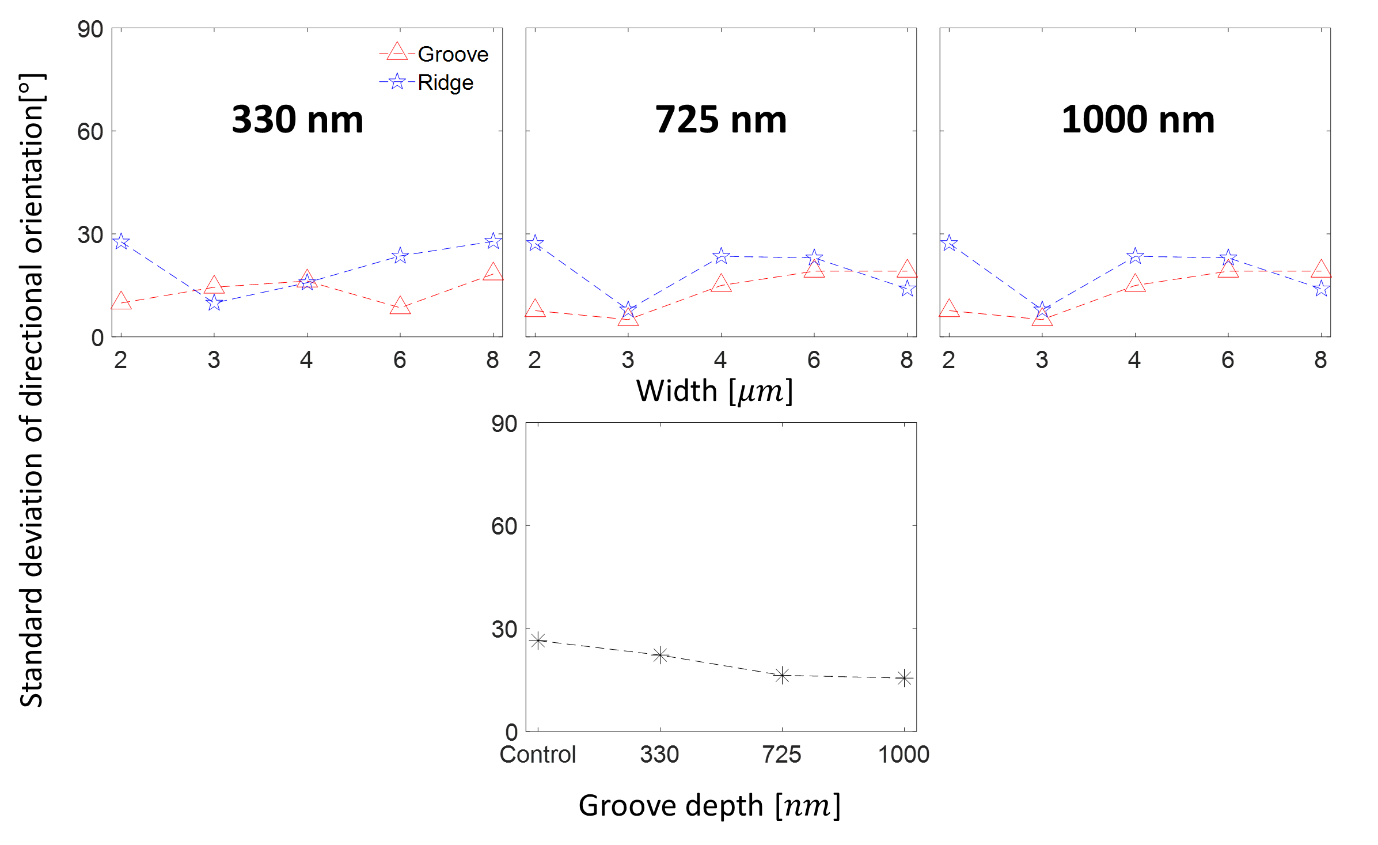


Figure S12. Standard deviation of cell degree alignment for varying ridge widths and groove widths with different groove depths (first row) and varying groove depths (second row).


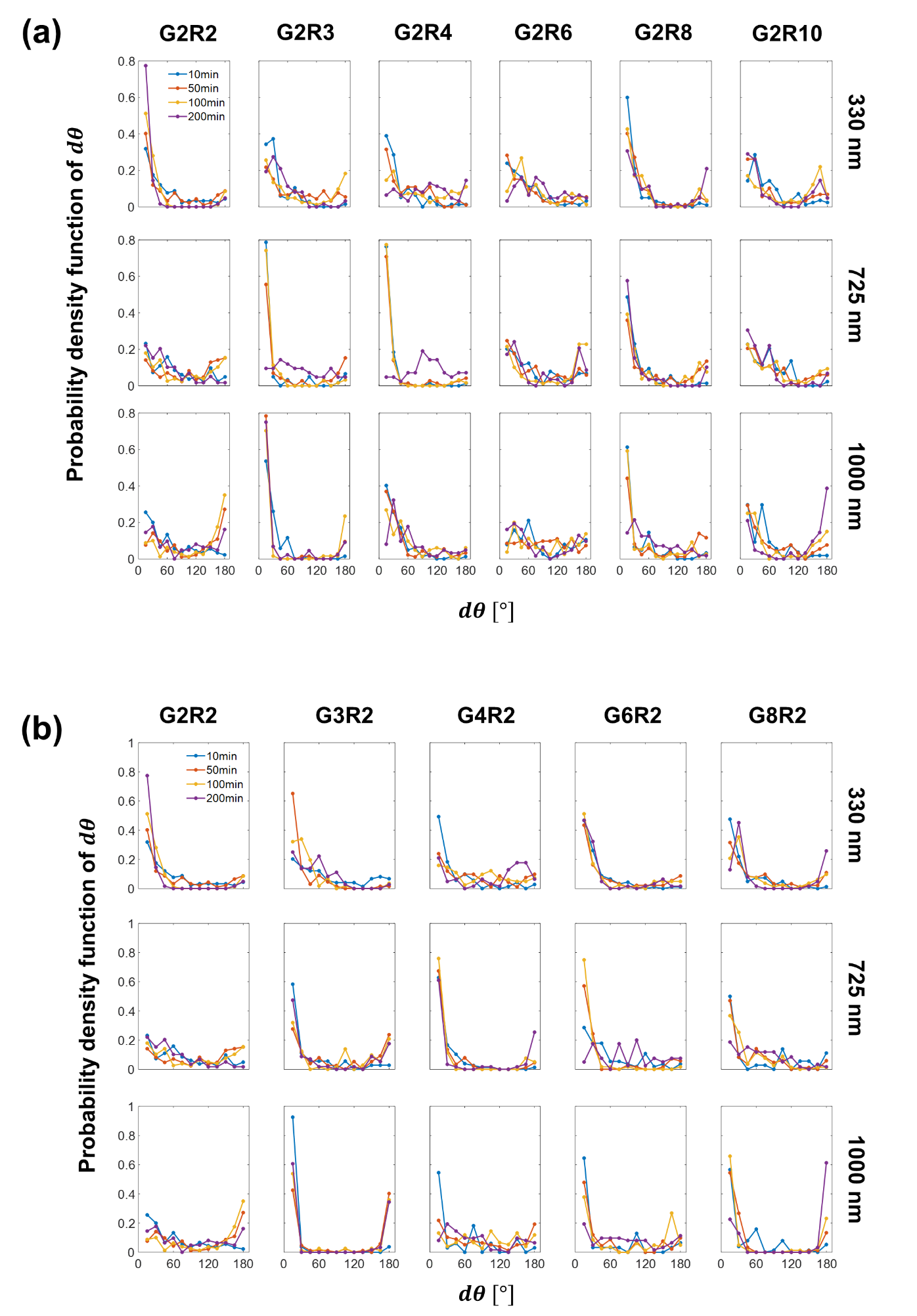


Figure S13. Probability density function of angular displacement ($d\theta$) for two different cases: G2RN (a) and GNR2 (b).


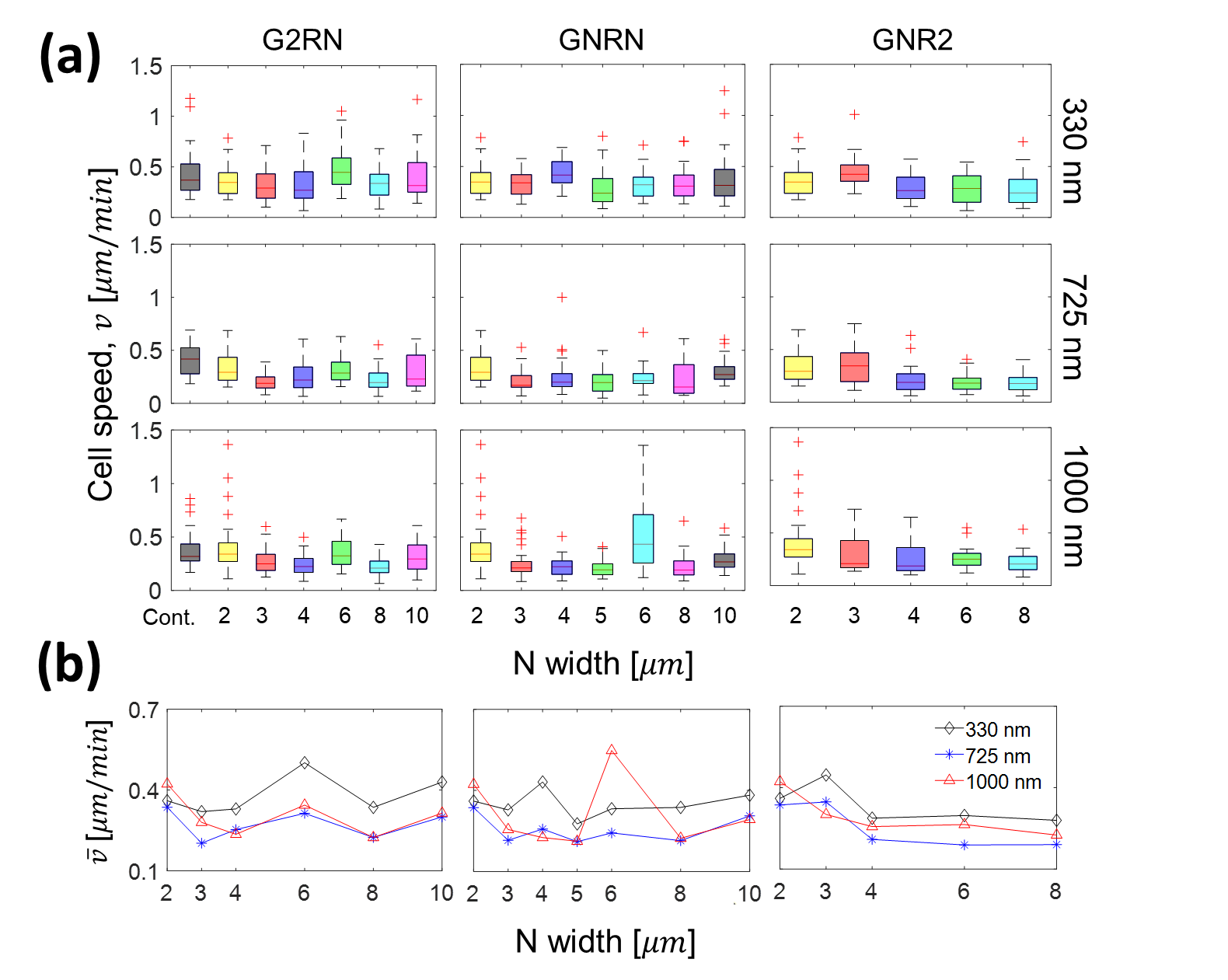


Figure S14. (a) Box-and-whisker diagrams of cell speed ($v$) with respect to individual topographic conditions. (b) The averaged cell speeds are plotted for different lateral conditions ($G_{w}$ and $R_{w}$).


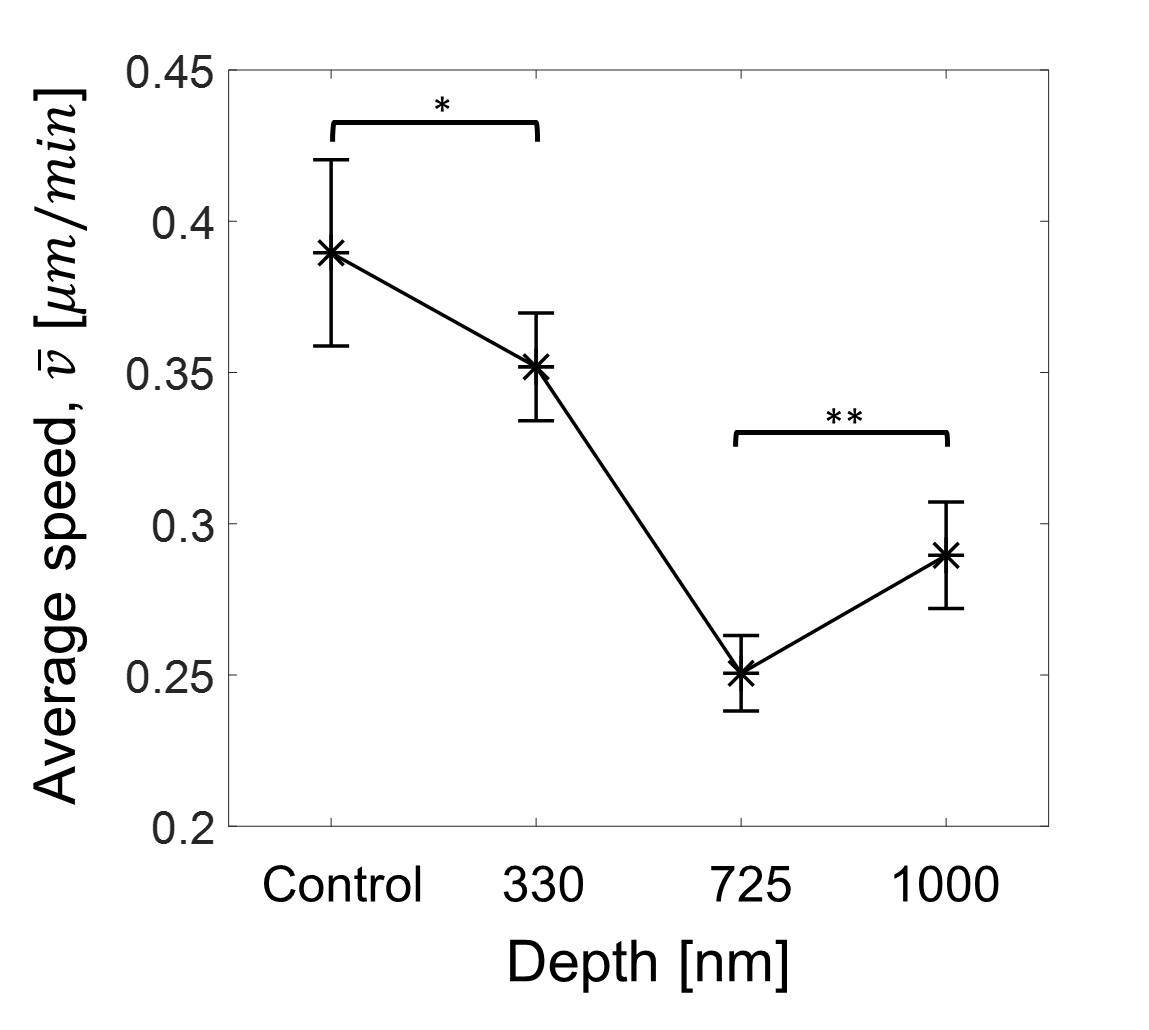


Figure S15. Cell migration speed with different groove depths. Kruskal Wallis test was performed to test for statistical significance (* p < 0.05 and ** p < 0.001). All error bars are mean $\pm$ standard error of mean (SEM).


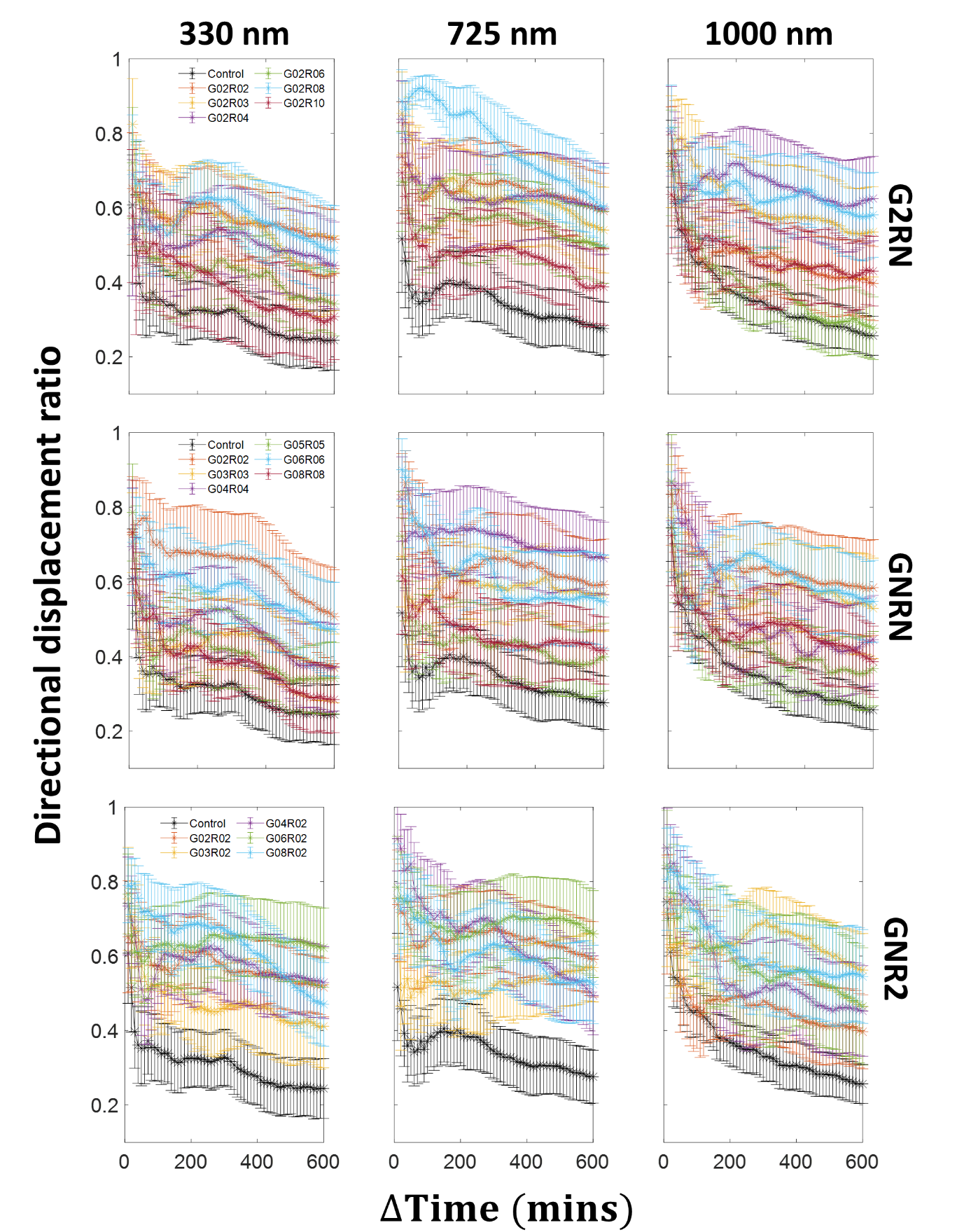


Figure S16. Directional displacement ratio (i.e., y direction moving length divided by the total travel length with error bars (mean $\pm$ standard error of mean (SEM) for the cells on all topographical cues as a function of the time interval ($\Delta$). Each row and column represent three different regions and depths scales, respectively.
